# Delineating SWI/SNF gene and subtype expression in cancer

**DOI:** 10.1186/s13072-026-00696-9

**Published:** 2026-08-20

**Authors:** Vilma Canfjorden, Malin Lindén, Tobias Österlund, Erik Larsson, Anders Ståhlberg

**Affiliations:** 1 Department of Laboratory Medicine, Sahlgrenska Center for Cancer Research, Institute of Biomedicine, Sahlgrenska Academy, University of Gothenburg, 413 90 Gothenburg, Sweden; 2 Department of Clinical Genetics and Genomics, Sahlgrenska University Hospital, Region Västra Götaland, 413 45 Gothenburg, Sweden; 3 Wallenberg Centre for Molecular and Translational Medicine, University of Gothenburg, 413 90 Gothenburg, Sweden; 4 Department of Medical Biochemistry and Cell Biology, Institute of Biomedicine, Sahlgrenska Academy, University of Gothenburg, 413 90 Gothenburg, Sweden; 5 Science for Life Laboratory, Institute of Biomedicine, University of Gothenburg, 413 90 Gothenburg, Sweden

**Keywords:** Cancer, cBAF, GBAF, Gene expression, PBAF, SWI/SNF, TCGA

## Abstract

**Background:**

Approximately 20% of all cancers contain mutations in genes coding for components of the SWI/SNF chromatin remodeling complex, which exists in three main subtypes: cBAF, PBAF, and GBAF. Mutations in SWI/SNF genes may disrupt chromatin remodeling and deregulate epigenetic processes, leading to aberrant gene expression profiles. However, the gene expression profiles of SWI/SNF genes and subtypes across different tumor types in relation to SWI/SNF mutations remain poorly understood.

**Results:**

In this study, we profile the expression levels of all 29 SWI/SNF genes in 33 tumor types using DNA and mRNA data from The Cancer Genome Atlas, comprising approximately 11,000 samples. We find that *ACTL6B*, *DPF1*, *DPF3* and *SMARCD3* vary the most among tumor types and that 10 out of 33 tumor types show distinct SWI/SNF gene expression signatures. The SWI/SNF gene expression in tumors are partly influenced by their tissue of origin. Furthermore, the mean expression levels of SWI/SNF subtype-specific genes are overall similar between tumor types, with higher variations in cBAF compared to PBAF and GBAF. Mutations in SWI/SNF genes result in only minor changes in SWI/SNF gene expression levels but are associated with downstream regulation of transcriptional programs linked to epigenetic regulation, especially in colon adenocarcinoma, kidney renal clear cell carcinoma, stomach adenocarcinoma and uterine corpus endometrial carcinoma.

**Conclusions:**

Our study provides insights into SWI/SNF gene and subtype expression in cancer, which may contribute to the development of biomarkers and targeted therapies.

**Supplementary Information:**

The online version contains supplementary material available at 10.1186/s13072-026-00696-9.

## Background

Cancer typically develops through mutations in oncogenes or tumor suppressor genes but can occur through different oncogenic mechanisms including but not limited to copy number alterations, chromosomal rearrangement, epigenetic mechanisms and viral oncogenesis. In the last decades, the importance of epigenetic processes in cancer development has become increasingly recognized [1]. Specifically, the SWItch/Sucrose Non-Fermentable (SWI/SNF) genes have been found to be frequently mutated in numerous cancer types, occurring in about 20% of all human cancers [2, 3]. The SWI/SNF chromatin remodeling complex utilizes energy from ATP hydrolysis to modify the epigenetic landscape and regulate gene expression by remodeling nucleosomes and exposing DNA, providing access to regulatory regions [4, 5]. The SWI/SNF complex can also regulate chromatin structure and gene expression antagonistically with the Polycomb repressive complex 2 (PRC2) that mediates gene repression through the histone modification H3K27me3 [6]. This opposition generates a transcriptional balance that is essential during development and lineage determination, exemplifying the importance of epigenetic regulation of gene expression [6–9].

**Fig. 1:**
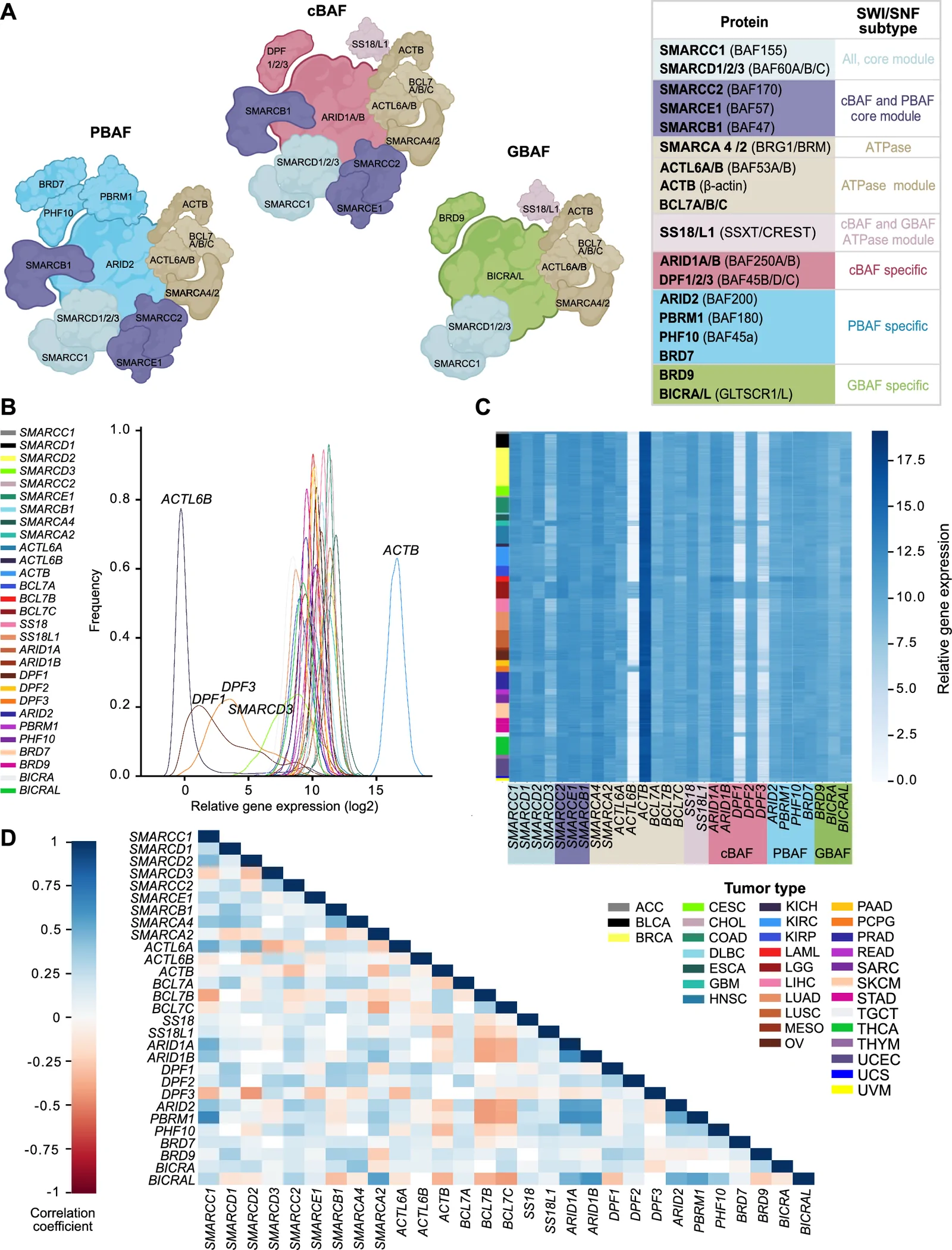
Expression of SWI/SNF genes in different tumor types. **A** Schematic illustration of SWI/SNF subtypes and genes. cBAF-specific subunits are shown in red, PBAF-specific subunits are shown in blue and GBAF-specific subunits are shown in green. Shared core components are shown in light blue, while core components shared by cBAF and PBAF only are shown in purple. The shared ATPase-module components are colored beige and the ATPase-module components shared between cBAF and GBAF are colored pink. (Created with https://BioRender.com). **B** Distribution and frequency of expression values for the 29 SWI/SNF genes. A kernel density estimation plot was used, n = 11,069. **C** Heatmap of SWI/SNF gene expression for all tumor samples. The SWI/SNF genes are grouped as in (**A**) and samples are sorted and color-coded vertically in relation to tumor type. **D** Pair-wise correlation analysis of expression between SWI/SNF genes. Spearman correlation coefficients for all gene pairs with *p* < 0.05 are shown, non-significant correlations are marked in white

The SWI/SNF complexes consist of 29 protein subunits, where some exist in different paralogs (Fig. 1A). The SWI/SNF subunits are assembled into different complex subtypes with diverse biochemical properties. The canonical BAF complex (cBAF), the polybromo BAF complex (PBAF), and the GLTSCR1/1L BAF complex (GBAF), also known as non-canonical BAF are the most characterized main subtypes [10, 11]. All subtypes share the structural core components SMARCC1 and one of the SMARCD paralogs (SMARCD1, SMARCD2 or SMARCD3) as well as one of the ATPase components, SMARCA2 or SMARCA4 [12, 13]. The ATPase module contains ACTL6A or ACTL6B, one of BCL7A, BCL7B or BCL7C and ACTB. Additionally, in cBAF and GBAF, SS18 or SS18L1 is present. Moreover, cBAF and PBAF contain the core components SMARCB1, SMARCE1 and SMARCC2. Some subunits are specific to each SWI/SNF subtype: ARID1A or ARID1B and DPF1, DPF2 or DPF3 in cBAF, and ARID2, PBRM1, PHF10 and BRD7 in PBAF. The GBAF complex specifically contains BRD9 and either BICRA or BICRAL (also known as GLTSCR1 and GLTSCR1L). Thus, compared to cBAF and PBAF, GBAF lacks SMARCB1, SMARCE1, SMARCC2, ARID and the DPF paralogs [3, 14]. Distinct SWI/SNF complexes have both specific and shared functions and genomic binding sites. cBAF primarily localizes to enhancers, PBAF is found at promoters and gene bodies, and GBAF mainly localizes to CTCF sites but is also found at both promoters and enhancers [11, 15–18]. The distinct SWI/SNF compositions are also believed to contribute with specificity in interactions with transcription factors and chromatin regulators [19]. Hence, SWI/SNF complexes are important in developmental cell transitions by controlling cell-type specific gene expression programs [20]. Specifically, the cBAF subtype plays a role in embryonic pluripotency, where it both activates and represses target genes [21–23], PBAF is involved in lineage-specific cell differentiation and cell type identity [22] and GBAF is important for maintaining the naïve pluripotency of embryonic stem cells [15].

Misregulation or mutation in SWI/SNF genes may result in tumor formation, where loss-of-function mutations are most common [4]. However, rather than inactivation of the entire complex, oncogenic activity has been shown to occur through deviant activity of the residual complexes [24, 25]. The core components *SMARCA4* and *SMARCB1* are important tumor suppressor genes [19, 26, 27]. However, the most frequently mutated gene in the SWI/SNF complex is the cBAF-specific *ARID1A*, which is mutated to varying extents in different tumor types, including about 45% of clear-cell ovarian and endometrioid cancers [2]. Mutations in the PBAF-specific *PBRM1* gene and overexpression of the cBAF-specific *DPF3* gene have been linked to kidney cancer, with *DPF3* overexpression altering gene expression through modifications in chromatin structure [28]. The GBAF-specific components have lower mutation rates compared to cBAF and PBAF, nevertheless *BICRA* and *BICRAL* have been shown to be important for cell proliferation in a prostate cancer cell line [10]. Non-mutational SWI/SNF expression differences may also cause cancer, as shown by epigenetic silencing of *SMARCA2*, aberrant splicing of *SMARCA4* and loss of protein expression of ARID1A or other SWI/SNF components [29–32]. Furthermore, post-translational modifications like acetylation can impact the function of SWI/SNF components, as reported for SMARCA2 [33]. Altogether, SWI/SNF complexes display key roles in cancer. However, the gene regulation of distinct SWI/SNF complexes and their subunits remains poorly understood, both across different tumor types and how mutations in SWI/SNF genes affect gene expression signatures.

In this study, we determine the mRNA expression levels of SWI/SNF genes and subtypes in cancer, linking their expression profiles to SWI/SNF mutations. We utilize publicly available datasets from The Cancer Genome Atlas Program (TCGA) [34, 35], including 11,069 samples from 33 different tumor types. We analyze the expression profiles of the 29 SWI/SNF genes and the three main subtypes, cBAF, PBAF and GBAF, across all tumor types and compare the expression with normal tissue [36]. We also compare gene expression signatures between samples with and without mutations in SWI/SNF genes and evaluate the impact of SWI/SNF mutations on patient overall survival.

## Methods

### Data pre-processing

To study the expression of all 29 genes in the SWI/SNF complex we utilized the TCGA database, including 11,069 samples generated from 33 tumor entities in the Pan-Cancer project [34, 35]. The data files were downloaded from the UCSC Xena Browser [37], including gene expression and mutation data (normalized mRNA expression dataset and MC3 public version, respectively). The gene expression matrix, i.e. TCGA Pan-Cancer Atlas cohort, contains preprocessed RNA sequencing expression values that were normalized and batch-effect corrected using the Pan-Cancer Atlas empirical Bayes (EB++) pipeline and presented as log-transformed values. Data analyses were performed on these processed expression values without further normalization. For the SWI/SNF gene expression analysis, values with no expression were replaced with the lowest detected value divided by two per gene to minimize the effect of non-continuous data.

### Gene expression analysis

We performed univariate analysis in Python v3.10.8, generating basic statistics of all 29 genes and tumor entities. The distribution of gene expression was performed with a kernel density estimation using Python v3.10.8 matplotlib v3.5.3, which was also utilized for generating the heatmaps. t-distributed stochastic neighbor embedding (t-SNE) plots were performed in Python v3.10.8 sklearn.manifold.TSNE v1.2.0 using default settings, including perplexity: 30, learning rate: auto and random state: 0. The t-SNE was used as dimensionality reduction method since it provided the best visualization of tumor groups compared to related methods. Hierarchical clustering of the gene expression data was performed using the Seaborn clustermap function, which applies agglomerative clustering by default. The correlation plots were generated using Python v3.10.8 with Spearman correlation and significance level of 0.05. The p-values were corrected for multiple testing using false discovery rate. The subtype-specific genes were divided into the three subtypes cBAF (*ARID1A*, *ARID1B*, *DPF1*, *DPF2* and *DPF3*), PBAF (*ARID2*, *PBRM1*, *PHF10* and *BRD7*) and GBAF (*BRD9*, *BICRA* and *BICRAL*). The relative subtype-specific gene expression was calculated as the mean gene expression for the subtype-specific genes per cancer type divided by the overall mean gene expression for each subtype for all cancer types. The results were ranked by highest to lowest value based on cBAF. To compare SWI/SNF subtypes between samples and tumor types, z-scores were used. Box plots of the z-scores were created for each cancer type using Python v3.10.8 plotly v5.15.0, visualizing the deviation from the overall mean expression of the subtypes. To compare expression of SWI/SNF components in tumor samples versus normal tissues, we utilized the TNMplot database, consisting of samples from GEO, GTEx, TCGA, and TARGET repositories [36]. In total, 22 of the 33 tumor types (ACC, LAML, BLCA, BRCA, COAD, ESCA, LIHC, LUAD, LUSC, OV, PAAD, PRAD, READ, KIRC, KICH, KIRP, SKCM, STAD, TGCT, THCA, UCS and UCEC) were represented in the TNMplot database.

### Mutation analysis

Mutation information was available for 9,102 samples in the TCGA database of which 8,767 samples also had gene expression data. This dataset was then divided into SWI/SNF gene mutated and non-mutated samples, resulting in 2,245 and 6,522 samples, respectively. Mutation metadata was formatted using bash in a UNIX environment and visualized using ggplot2 v3.4.2, a data visualization package from R v4.3.0, and plotly v5.15.0 in python. Mutation annotations for the 29 SWI/SNF genes from the MC3 dataset (version 0.2.8) were analyzed across mutation categories. The impact of the top five mutation types on SWI/SNF gene expression was analyzed using one-way ANOVA with post hoc Dunnett’s tests followed by Benjamini–Hochberg correction for multiple testing.

### Differential expression analysis and enrichment analysis

To determine significantly regulated genes between each distinct tumor type with a SWI/SNF signature compared to the remaining 23 tumor types and between mutated and non-mutated tumor samples, the Mann–Whitney U test was used adjusting p-values with Benjamini–Hochberg correction for multiple testing. Significant regulated genes were selected based on fold change > 2 and false discovery rate < 0.05 and used for enrichment analysis. The enrichment analysis was conducted using clusterProfiler v4.8.3 in R v4.3.0 with the gene set Chemical and Genetic Perturbations (versions 7.5.1). Statistical significance was determined using adjusted p-values with the Benjamini–Hochberg false discovery rate correction method. Kaplan–Meier survival estimates were performed in Python using KaplanMeierFitter (lifelines package) and in R v 4.3.2 using the survival package with overall survival status and time obtained from the clinical information in the TCGA Xena file. The log-rank test and cox proportional hazards model was used to assess significant differences between SWI/SNF mutated and non-mutated groups for groups with more than 10 samples.

## Results

### Characterization of SWI/SNF gene expression in 33 tumor types

To assess the gene expression of all 29 SWI/SNF components in cancer (Fig. 1A), we utilized data from the TCGA database, including 33 different tumor types and 11,069 samples (Table 1, Additional file 1 and Supplementary Table 1). The RNA sequencing data set was normalized and batch-corrected to enable comparisons between samples and tumor types. The gene expression of individual SWI/SNF genes varied both between and within tumor types (Fig. 1B, C). The expression of *DPF1*, *DPF3* and *SMARCD3* varied substantially among samples and tumor types, while the other genes showed less variability. Absolute gene expression levels cannot be directly assessed due to unknown reverse transcription yield and amplification efficiency between target sequences. However, at a qualitative level, *ACTB* was expressed at the highest level, while *ACTL6B*, *DPF1*, *DPF3* and *SMARCD3* showed lower expression compared to the other 24 genes.

**Table 1:** Study overview with tumor types, number of samples and mutation frequency

Abbreviation	Tumor type	Number of samples	Mutation frequency (%)
ACC	Adrenocortical carcinoma	79	7.6
BLCA	Bladder urothelial carcinoma	427	54.7
BRCA	Breast invasive carcinoma	1215	13.6
CESC	Cervical squamous cell carcinoma and endocervical adenocarcinoma	309	26.9
CHOL	Cholangiocarcinoma	45	44.4
COAD	Colon adenocarcinoma	492	39.7
DLBC	Lymphoid neoplasm diffuse large B-cell lymphoma	48	40.5
ESCA	Esophageal carcinoma	196	25.7
GBM	Glioblastoma multiforme	168	5.3
HNSC	Head and neck squamous cell carcinoma	566	22.4
KICH	Kidney chromophobe	91	4.5
KIRC	Kidney renal clear cell carcinoma	606	48.1
KIRP	Kidney renal papillary cell carcinoma	323	23.6
LAML	Acute myeloid leukemia	173	n/a
LGG	Brain lower grade glioma	533	14.3
LIHC	Liver hepatocellular carcinoma	424	28.5
LUAD	Lung adenocarcinoma	576	36.5
LUSC	Lung squamous cell carcinoma	553	32.6
MESO	Mesothelioma	87	9.9
OV	Ovarian serous cystadenocarcinoma	309	14.3
PAAD	Pancreatic adenocarcinoma	183	8.2
PCPG	Pheochromocytoma and paraganglioma	187	0.6
PRAD	Prostate adenocarcinoma	550	6.7
READ	Rectum adenocarcinoma	170	18.0
SARC	Sarcoma	265	8.5
SKCM	Skin cutaneous melanoma	473	43.2
STAD	Stomach adenocarcinoma	450	41.3
TGCT	Testicular germ cell tumors	139	1.6
THCA	Thyroid carcinoma	572	3.1
THYM	Thymoma	122	5.9
UCEC	Uterine corpus endometrial carcinoma	555	58.5
UCS	Uterine carcinosarcoma	57	21.1
UVM	Uveal melanoma	80	3.8

Total number of samples with gene expression profile, n = 11,069. Mutation frequency is calculated from the subset of samples with both gene expression data and mutation information, n = 8,767

*n/a* no mutation information

We performed pairwise correlation analysis between the expression of all SWI/SNF genes across all samples and found that most observed pairwise correlations (93%) were significant (Fig. 1D and Supplementary Table 2). The *ARID* genes (*ARID1A*, *ARID1B* and *ARID2*) were negatively correlated with *BCL7B* and *BCL7C*, but positively correlated with *BICRAL*, *PBRM1* and *SMARCC1*. Interestingly, *ARID* genes were positively correlated with each other despite that each SWI/SNF complex can only contain one of the translated proteins. *BCL7B* and *BCL7C* were also negatively correlated with *BICRAL* and *PBRM1*. The cBAF-specific component *DPF3* was negatively correlated with *SMARCC1*, *SMARCD2* and *ACTL6A*, which are part of the core or ATPase module. *DPF1*, *DPF2* and *DPF3* are paralogs, with only one present in each cBAF complex. The lower expression of *DPF1* and *DPF3* indicates a higher prevalence of cBAF subtypes containing *DPF2*. GBAF-specific genes *BRD9*, *BICRA* and *BICRAL* generally showed positive correlations with components absent in GBAF complexes, such as *SMARCC2*, *SMARCE1* and *SMARCB1*, as well as cBAF-specific and PBAF-specific components. Notably, certain SWI/SNF paralogs, such as *BICRA/BICRAL*, *SMARCA4/SMARCA2*, *SMARCD1/SMARCD2* and *SS18/SS18L1* displayed weak correlations. In conclusion, no clear competition between SWI/SNF components were found at gene expression level, i.e., no strong negative correlations between SWI/SNF paralogs or subunits present in different complexes. Instead, these genes were either weakly correlated or positively correlated, as in the case of *ARID* genes, suggesting that the mutual exclusivity observed during protein complex assembly was not reflected at the gene expression level.

### Certain tumor types have distinct SWI/SNF gene expression signatures

**Fig. 2:**
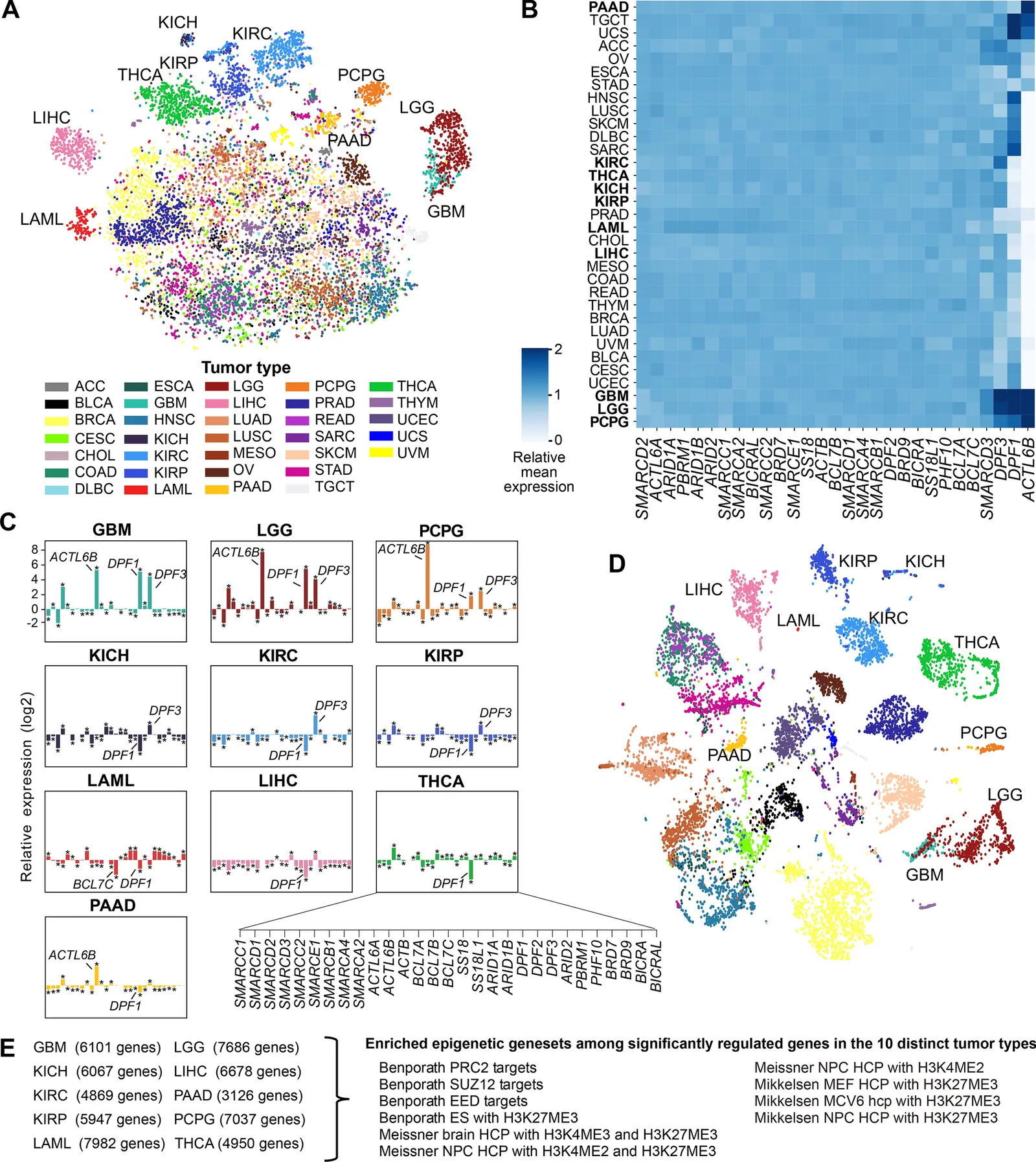
SWI/SNF gene expression patterns in different tumor types.** A** t-SNE plot based on gene expression of all 29 SWI/SNF genes, colored by tumor type. The most distinct tumor types are indicated, including acute myeloid leukemia (LAML), liver hepatocellular carcinoma (LIHC), kidney chromophobe (KICH), thyroid carcinoma (THCA), kidney renal clear cell carcinoma (KIRC), kidney renal papillary cell carcinoma (KIRP), pancreatic adenocarcinoma (PAAD), pheochromocytoma and paraganglioma (PCPG), brain lower grade glioma (LGG) and glioblastoma multiforme (GBM). **B** Heatmap of mean expression of each SWI/SNF gene in each tumor type relative to the overall mean value for each gene in all tumor types. Genes and tumor types are grouped by hierarchical clustering. The most distinct tumor types in (**A)** are marked in bold. **C** Relative gene expression of SWI/SNF genes for each tumor type with distinct t-SNE profile compared to the mean of each gene in remaining 23 tumor types. The genes with most divergent expression are indicated. * Significant using Mann–Whitney U test with Benjamini–Hochberg correction for multiple testing and adjusted *p* < 0.05. **D** t-SNE plot based on gene expression for the whole transcriptome. In total, 20,533 genes and 11,069 samples were profiled. The tumor types with distinct SWI/SNF gene signatures identified in (**A)** are marked in bold. **E** Summary of enriched gene sets related to epigenetic regulation based on functional enrichment analysis using Chemical and Genetic Perturbation gene set collections for tumor entities with SWI/SNF signatures. The number of significantly regulated genes for each distinct tumor type with a SWI/SNF signature compared to the remaining 23 tumor types are indicated, based on fold change cutoff > 2 and Mann–Whitney U test with Benjamini–Hochberg correction for multiple testing with adjusted *p* < 0.05. The functional enrichment for each tumor type is shown in Supplementary Fig. 3, Additional file 1

To assess the overall SWI/SNF gene expression signature, we performed t-distributed stochastic neighbor embedding (t-SNE) analysis based on the expression levels of all 29 SWI/SNF genes (Fig. 2A and Supplementary Figs. 1, 2, Additional file 1). Ten tumor types exhibited distinct signatures compared to the other 23 entities, including acute myeloid leukemia, liver hepatocellular carcinoma, kidney chromophobe, thyroid carcinoma, kidney renal papillary cell carcinoma, kidney renal clear cell carcinoma, pancreatic adenocarcinoma, pheochromocytoma and paraganglioma, brain lower-grade glioma and glioblastoma multiforme. The remaining tumor types showed overlapping gene expression profiles, although minor subgroupings related to specific tumor types were also observed within the main cluster. We compared tumor types with distinct SWI/SNF expression patterns to the remaining tumor types, identifying *ACTL6B*, *DPF1* and *DPF3* as the most variable genes (Fig. 2B, C). Heatmap visualization of the relative mean expression for each tumor type showed that cancers originating from the brain or nervous tissue (glioblastoma multiforme, brain lower-grade glioma as well as pheochromocytoma and paraganglioma) expressed relatively high levels of *ACTL6B*, *DPF1*, *DPF3* and *SMARCD3* (Fig. 2B, C and Supplementary Figs. 1, 2, Additional file 1). Kidney-related and thyroid tumor types (kidney renal clear cell carcinoma, kidney chromophobe, kidney renal papillary cell carcinoma and thyroid carcinoma) grouped together, with low *DPF1* expression and high *DPF3* expression. When the whole transcriptome was analyzed, samples from the same tumor type clustered together, including the 10 entities with specific SWI/SNF signatures (Fig. 2D). We then identified all regulated genes for each of the 10 tumor types with distinct SWI/SNF patterns compared to all the other 23 tumor types ( Supplementary Table 3). Functional gene enrichment analyses showed that epigenetic regulation was consistently enriched across multiple cancer types with SWI/SNF signatures (Fig. 2E and Supplementary Fig. 3, Additional file 1). In summary, while SWI/SNF gene expression varied across and within tumor types, 10 out of 33 tumor types exhibited distinct SWI/SNF expression profiles, with *ACTL6B*, *DPF1*, *DPF3* and *SMARCD3* showing the most pronounced differences.

### Tumors partly retain SWI/SNF gene expression patterns from their tissue of origin

**Fig. 3:**
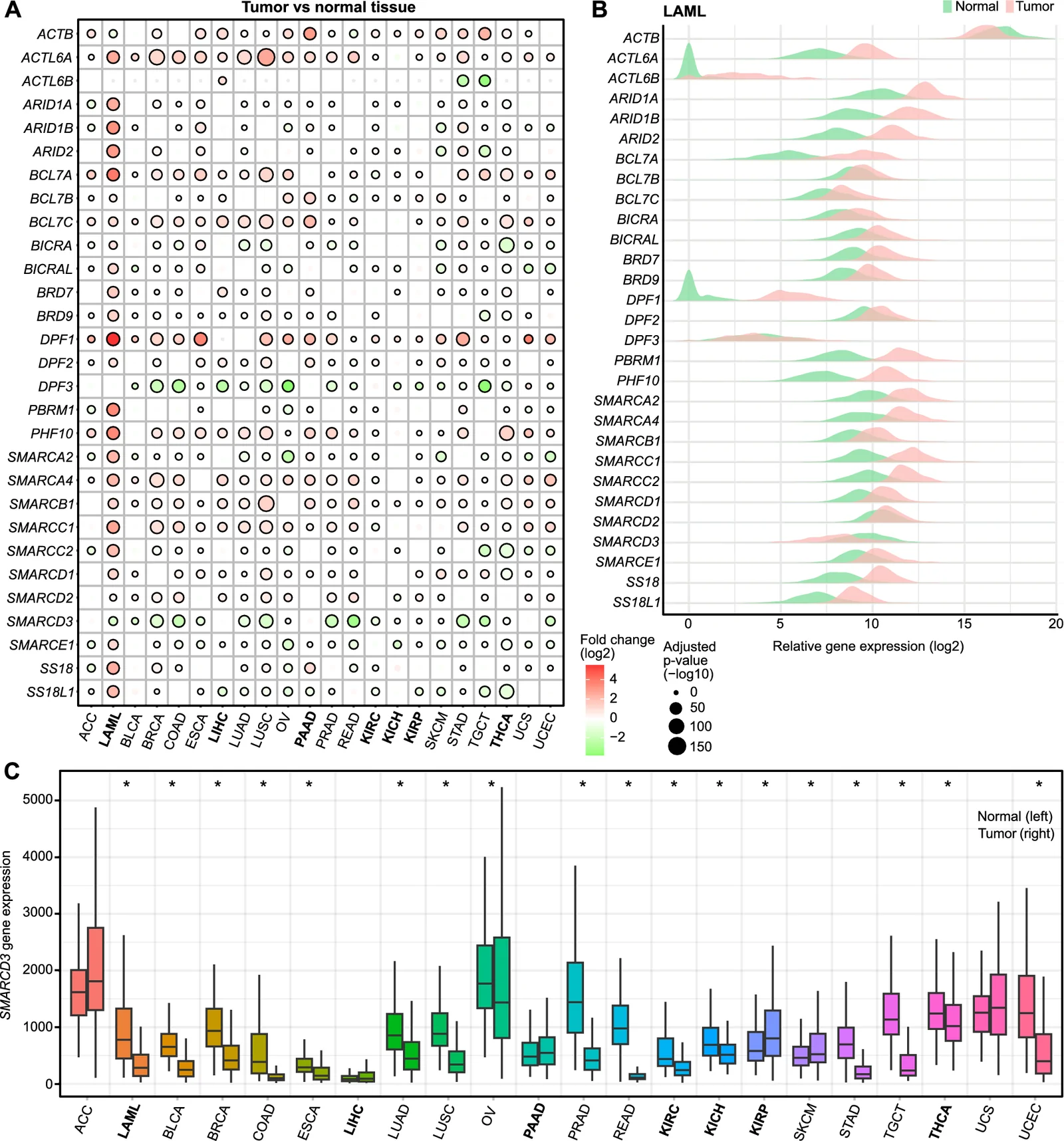
Expression of SWI/SNF genes in tumor and normal tissue. **A** Dot plot visualizing relative expression of SWI/SNF genes in tumor compared to normal tissue based on the TNMplot database. Color indicates fold change and dot size indicates the adjusted *p*-value using Mann–Whitney U test with Benjamini–Hochberg correction for multiple testing. **B** Density plots of SWI/SNF gene expression in normal and tumor tissues based on the TNMplot database in acute myeloid leukemia (LAML). Additional examples are shown in Supplementary Fig. 4, Additional file 1. **C** Box plot of *SMARCD3* gene expression in normal and tumor samples based on the TNMplot database. *Significant using Mann–Whitney U test with *p* < 0.05 and a minimum expression of 10 per group. Additional examples are shown in Supplementary Fig. 5, Additional file 1. The tumor types with distinct SWI/SNF gene signatures identified in Fig. 2A are marked in bold

To discern if the SWI/SNF expression patterns are influenced by the cell type, we compared the expression in tumor samples with normal tissues using the TNMplot database [36]. Many SWI/SNF genes displayed similar expression levels in tumor and normal tissues (Fig. 3A–C and Supplementary Figs. 4, 5, Additional file 1). However, there were also large variations in certain tumor types for different SWI/SNF components. For the 7 out of 10 tumor types with distinct SWI/SNF expression profiles present in the TNMplot database, we observed that acute myeloid leukemia displayed increased expression of most SWI/SNF components in tumor samples, while the kidney cancers were similar to normal tissue (Fig. 3A, B and Supplementary Fig. 4, Additional file 1). Furthermore, the expression of SWI/SNF components *ACTL6B*, *DPF1*, *DPF3* and *SMARCD3,* that contributed most to the diverse SWI/SNF expression profiles, were also influenced by the tissue of origin. For example, in kidney chromophobe distinguished by relatively high *DPF3* and low *DPF1* expression compared to most other tumor types, the expression in normal tissues were even higher for *DPF3* and lower for *DPF1* (Supplementary Fig. 4, Additional file 1). Several individual SWI/SNF genes exhibited diverging expression patterns between tumor and normal tissues. *ACTL6A* and *DPF1* consistently showed increased expression in tumor samples, while *SMARCD3* and *DPF3* showed increased expression in normal tissues (Fig. 3A–C and Supplementary Fig. 5, Additional file 1). Interestingly, the enzymatic paralogs *SMARCA4* and *SMARCA2* showed diverse expression patterns. In general, the expression of *SMARCA4* was increased and *SMARCA2* decreased in tumor tissue, which is consistent with the low levels of SMARCA2 observed in undifferentiated cells [23, 38]. Together, these data indicate that tumors partly retain SWI/SNF expression patterns from the normal tissue they originate from, although substantial variations in expression levels were observed.

### SWI/SNF subtype expression is stable between tumor entities

**Fig. 4:**
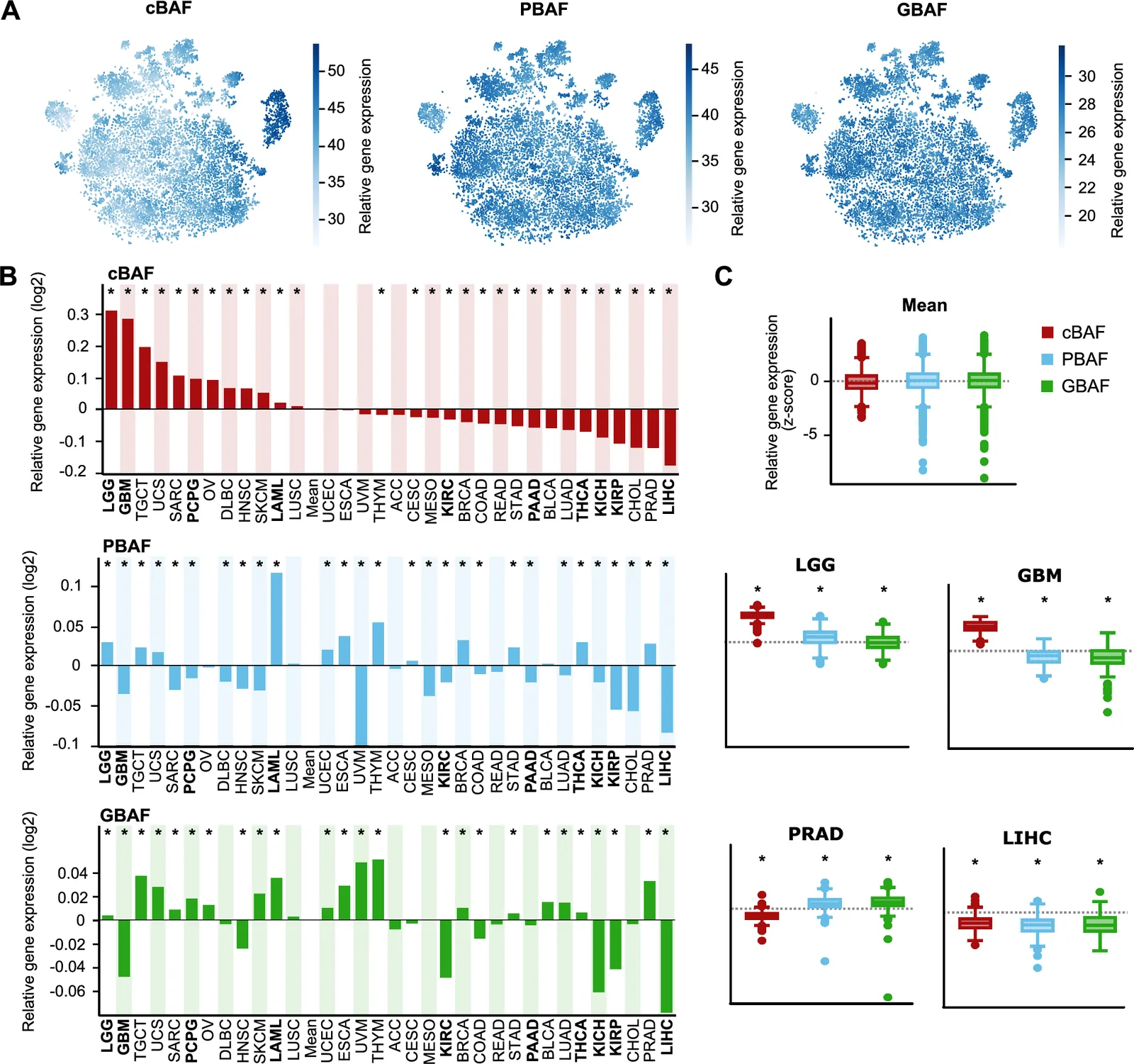
Expression of SWI/SNF subtypes in different tumor types.** A** Relative expression of cBAF-, PBAF- and GBAF-specific genes shown in the t-SNE plot based on gene expression of all 29 SWI/SNF genes. The scale bar represents the relative expression calculated as the sum of all subtype-specific genes. **B** Relative gene expression of each SWI/SNF subtype per tumor type compared to the mean subtype expression for all tumor types. Tumor types are ranked based on cBAF expression. Tumor types with distinct SWI/SNF signatures are shown in bold. * Significant using Student’s t-test with Benjamini–Hochberg correction for multiple testing and adjusted *p* < 0.05. **C** Box plot of relative cBAF, PBAF and GBAF expression across different tumor types. Z-scores for selected entities are shown, where Mean represents data for all tumor types. Box plots for remaining tumor types are shown in Supplementary Fig. 6, Additional file 1. The dotted line indicates the mean expression level. * Significant using Student’s t-test with Benjamini–Hochberg correction for multiple testing and adjusted *p* < 0.05

To determine the gene expression signatures of the three main SWI/SNF subtypes, cBAF, PBAF and GBAF, we calculated the total expression of the subtype-specific genes and visualized their expression in the t-SNE plot based on all SWI/SNF genes (Fig. 4A). We next investigated the expression of cBAF-, PBAF- and GBAF-specific genes in all 33 tumor types (Fig. 4B, C and Supplementary Fig. 6, Additional file 1). Overall, the level of cBAF varied more between tumor types than PBAF and GBAF, where brain lower-grade glioma and glioblastoma multiforme displayed highest cBAF expression, while prostate adenocarcinoma and liver hepatocellular carcinoma showed lowest cBAF expression. Among the 10 tumor types with distinct SWI/SNF signature, pheochromocytoma and paraganglioma also showed a somewhat elevated cBAF level, while the remaining tumor types had a lower expression of cBAF. The variations in PBAF and GBAF were low across the 33 tumor entities (Fig. 4B). Among the 10 tumor types with distinct SWI/SNF signature, acute myeloid leukemia displayed increased PBAF and GBAF expression while the four kidney and liver tumor types displayed decreased expression of all three subtypes. In summary, the expression levels of SWI/SNF subtypes were overall similar between tumor types with somewhat higher variations observed for cBAF.

### Mutations in SWI/SNF genes have minor effects on SWI/SNF gene expression

**Fig. 5:**
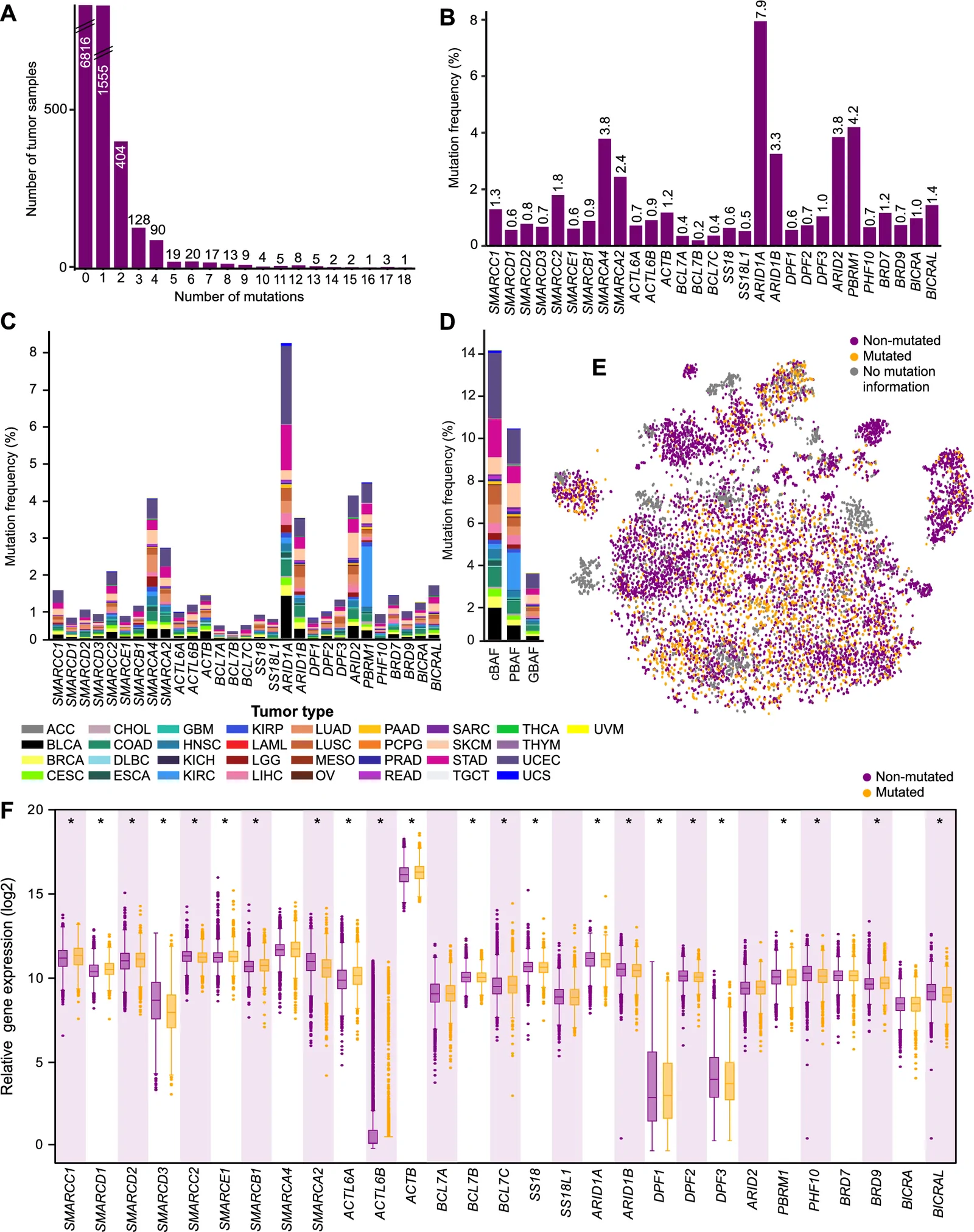
SWI/SNF mutations and impact on SWI/SNF gene expression.** A** Number of SWI/SNF mutations in each tumor sample, n = 9102. **B** Mutation frequency of each SWI/SNF gene in all tumor types, n = 9102. **C** Mutation frequency for each SWI/SNF gene in relation to tumor type, n = 9102. For detailed information, see Supplementary Table 4. **D** Mutation frequency for each SWI/SNF subtype in relation to tumor type, n = 9,102. **E** t-SNE plot based on gene expression of all SWI/SNF genes, where mutated (orange), non-mutated (purple) and unknown (grey) samples are marked, n = 11,069. **F** Boxplot comparing the gene expression of non-mutated samples (purple) and mutated samples (orange) per SWI/SNF gene. *Significant using Welch t-test with Benjamini–Hochberg correction for multiple testing < 0.05

To examine the effects of mutations in SWI/SNF genes on expression signatures, we compared the mutation status of each sample with its transcriptomic profile, yielding data for 9,102 samples (Fig. 5A). Overall, 25% of the samples had at least one mutation in a SWI/SNF gene, ranging from 1 to 19 mutations per sample where 109 samples contained more than four SWI/SNF mutations. Uterine corpus endometrial carcinoma and bladder urothelial carcinoma displayed the highest frequency of tumors with SWI/SNF mutations (> 50%), while pheochromocytoma, paraganglioma and testicular germ cell tumors showed the lowest frequency (around 1%) (Table 1 and Supplementary Table 4). We found that *ARID1A* was the most frequently mutated SWI/SNF gene (7.9%), followed by *PBRM1* (4.2%), *SMARCA4* (3.8%) and *ARID2* (3.8%) (Fig. 5B and Supplementary Table 4). Tumor-specific mutation patterns emerged, for example with *ARID1A* mutated in 44% of uterine corpus endometrial carcinomas, *PBRM1* in 40% of kidney renal clear cell carcinomas and *ARID2* in 14% of ovarian serous cystadenocarcinomas (Fig. 5C and Supplementary Table 4). cBAF-specific genes were most frequently muted (14%), followed by PBAF (9.9%) and GBAF (3.1%) (Fig. 5D). The most common types of SWI/SNF mutations were missense (58%), nonsense (14%) and frameshift deletion (12%), with the majority of missense mutations categorized as probably or possibly damaging (Supplementary Fig. 7, Additional file 1 and Supplementary Tables 4, 5, Additional files 5, 6).

Despite the high mutation frequency in certain SWI/SNF genes, there were no major differences between mutated and non-mutated samples in the t-SNE plot based on SWI/SNF genes (Fig. 5E). We compared the average expression of each SWI/SNF gene for all mutated versus non-mutated samples and found that *SMARCD3*, *SMARCA2* and *ACTL6B* were significantly downregulated more than 20% (Fig. 5F and Supplementary Table 6). Interestingly, when evaluating each tumor type separately, *SMARCD3* and *DPF3* were the only genes except *ACTL6B* that was significantly regulated more than 20% in several tumor types (Supplementary Table 6). *SMARCD3* was downregulated in kidney renal clear cell carcinoma, lung adenocarcinoma, stomach adenocarcinoma, uterine corpus endometrial carcinoma and uterine carcinosarcoma, while *DPF3* was upregulated in mutated samples in breast invasive carcinoma and kidney renal clear cell carcinoma, and downregulated in liver hepatocellular carcinoma and uterine corpus endometrial carcinoma. We then compared the SWI/SNF genes with significant differential expression with their expression in tumor and normal tissues (Supplementary Fig. 4, Additional file 1). For example, *SMARCD3* and *DPF3* exhibited significant decreased expression in both SWI/SNF-mutated compared to non-mutated samples and in tumor compared to normal tissues in kidney renal clear cell carcinoma and liver hepatocellular carcinoma, respectively, indicating that SWI/SNF mutations may shift SWI/SNF gene expression levels away from the levels observed in their tissue of origin. We then evaluated the impact of the top five mutation types, including missense, nonsense, frameshift deletion, silent and splice site on SWI/SNF gene expression levels (Supplementary Table 6). Most strikingly, samples with missense mutations in SWI/SNF genes displayed significant downregulation of *SMARCD3* and *DPF3* expression compared to non-mutated samples, while samples with nonsense or frameshift deletions had significant downregulation of *SMARCA4* and *BRD7* expression. In summary, SWI/SNF genes were mutated in a tumor-type-specific manner. However, mutations in SWI/SNF genes caused only minor regulatory effects on SWI/SNF gene expression levels.

### Mutations in SWI/SNF genes impact global gene expression and survival outcomes

**Fig. 6:**
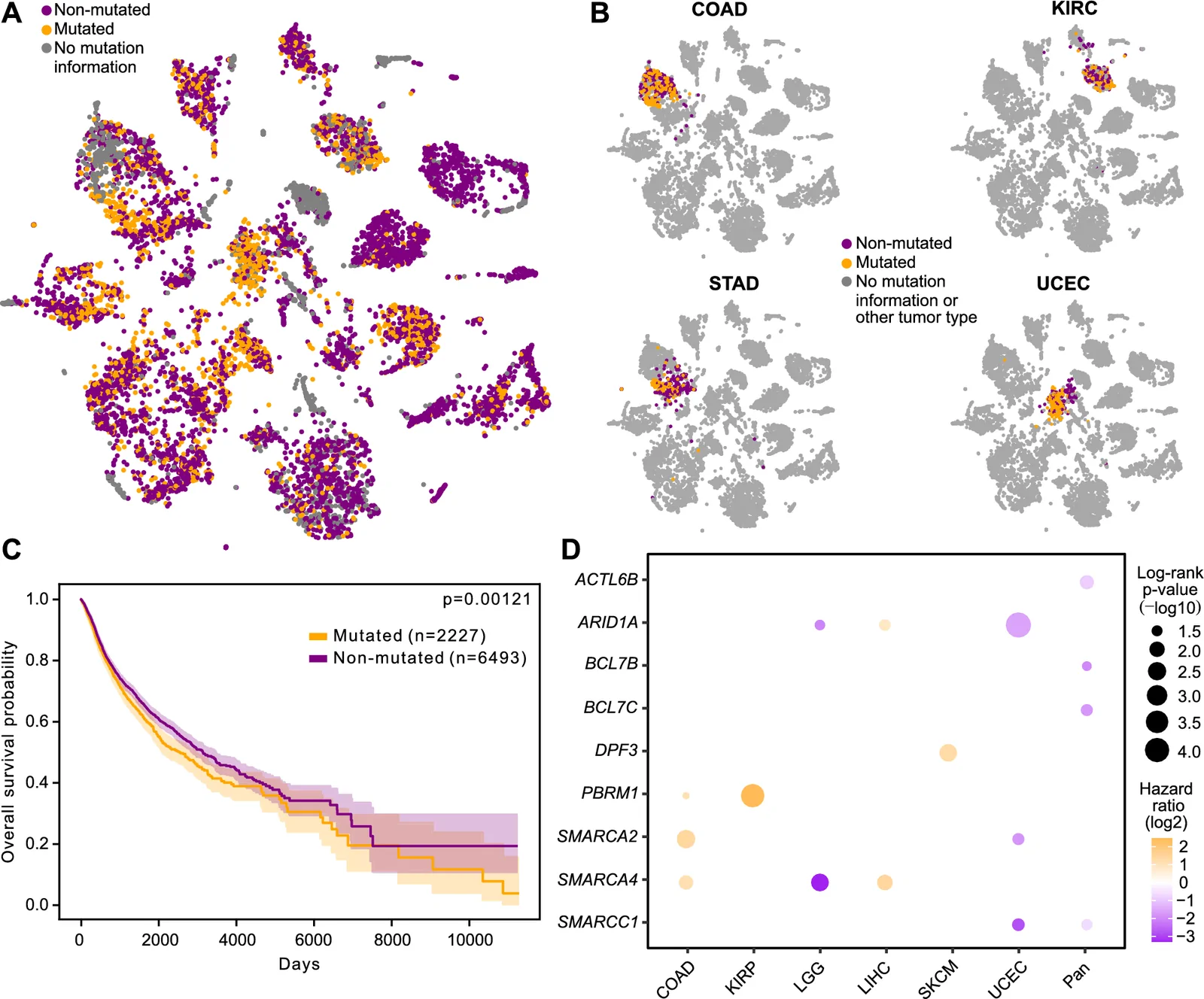
Effect of SWI/SNF mutations on global gene expression and patient survival. **A** t-SNE plot based on gene expression of the whole transcriptome, where mutated (orange), non-mutated (purple) and unknown (grey) samples are marked. n = 11,069. **B** t-SNE plot based on gene expression of the whole transcriptome, where mutated (orange) and non-mutated samples (purple) are marked for colon adenocarcinoma (n = 492), kidney renal clear cell carcinoma (n = 606), stomach adenocarcinoma (n = 450) and uterine corpus endometrial carcinoma (n = 555). Grey represents either another tumor type or samples without any tumor information. **C** Kaplan–Meier plot visualizing overall survival pan-cancer of patients with tumors that have a SWI/SNF mutation compared to non-mutated. Log-rank *p*-values and 95% confidence interval are shown **D** Dot plot visualizing significant overall survival differences for patients with mutation in individual SWI/SNF genes in specific tumor types or pan-cancer, based on log-rank test and Cox hazard ratio. Only gene and tumor type pairs with log-rank *p* < 0.05 and a minimum of 10 samples per group are shown

Next, we analyzed the impact of SWI/SNF mutations on the entire transcriptome. Samples primarily grouped by tumor type, rather than by mutation status (Fig. 6A). However, distinct internal subgroups based on mutation status were clearly observed for tumor types with high SWI/SNF mutation frequencies, such as colon adenocarcinoma, kidney renal clear cell carcinoma, stomach adenocarcinoma and uterine corpus endometrial carcinoma (Fig. 6B and Supplementary Fig. 8, Additional file 1). We performed differential gene expression analysis comparing mutated with non-mutated samples for each tumor type. Sixteen tumor types had significantly regulated genes (Supplementary Table 7, Additional file 8), where colon adenocarcinoma (218 genes), kidney renal clear cell carcinoma (276 genes), lung adenocarcinoma (54 genes), skin cutaneous melanoma (33 genes), stomach adenocarcinoma (339 genes) and uterine corpus endometrial carcinoma (416 genes) displayed highest number of regulated genes. Functional enrichment analysis of each of these tumor types revealed terms linked to epigenetic regulation, such as H3K27me3 modifications and PRC2 complexes (Supplementary Fig. 9, Additional file 1).

We then investigated if mutations in SWI/SNF genes were associated with survival outcomes. SWI/SNF mutations were significantly associated with decreased overall survival in pan-cancer, lung adenocarcinoma and rectum adenocarcinoma, but with increased overall survival in stomach adenocarcinoma and uterine corpus endometrial carcinoma (Fig. 6C and Supplementary Fig. 10, Additional file 1). Next, we evaluated the prognostic impact of individual SWISNF mutations in each tumor type and pan-cancer (Fig. 6D and Supplementary Table 8). There were few significant differences in survival for individual SWI/SNF genes; out of 986 tests (34 tumor types including pan-cancer, and 29 genes), 16 showed significant differences in overall survival. For example, mutations in *PBRM1* were associated with decreased overall survival in kidney renal papillary cell carcinoma, while mutations in *ARID1A* and *SMARCA4* were associated with increased overall survival in uterine corpus endometrial carcinoma and brain lower grade glioma, respectively. In summary, SWI/SNF mutations regulated downstream transcriptional profiles linked to epigenetic processes. Furthermore, a few SWI/SNF mutations were associated with overall survival outcomes in specific tumor types, indicating context-specific functions of individual SWI/SNF components.

## Discussion

The SWI/SNF chromatin remodeling complex alters specific genomic regions, allowing them to become accessible for biological processes, such as transcription, replication and DNA repair. The fact that SWI/SNF genes are mutated in 20% of human cancers suggests important functions in cancer development and progression. In this study, we analyzed SWI/SNF gene expression across 33 tumor types using the TCGA dataset.

Many SWI/SNF components are evolutionary conserved among species [39], highlighting the critical role of SWI/SNF in fundamental cellular functions, such as chromatin organization and transcriptional activity. We observed that the expression of most SWI/SNF genes and specific SWI/SNF subtypes was highly similar across tumor types. However, the diversity of mammalian SWI/SNF complexes suggest that different complexes and components have specific and non-redundant functions, allowing specific transcriptional regulation across developmental stages and cell types, due to the contribution of varying subunits [20, 40]. Our data analysis showed that the expression of several SWI/SNF genes vary within and between tumor types. Unexpectedly, we observed few negative correlations between SWI/SNF genes, even between subunit paralogs or subtype-specific components, suggesting that there is little competitive regulation at gene level unlike the reported competition between protein paralogs. It is known that the SWI/SNF composition is controlled by expression and combinatorial assembly of SWI/SNF components and their paralogs at protein level [40] and that changes in the amount of available paralogs can lead to subunit switching, thereby affecting the function of the complex [23, 41–43]. Our comparison between tumor and normal tissues suggests that the SWI/SNF gene expression in tumor samples are in part influenced by the cell type and tissue of origin, even though distinct differences in expression profiles between tumor and normal samples were observed.

We identified 10 tumor types with distinct SWI/SNF gene signatures where variable expression of *ACTL6B*, *DPF1*, *DPF3* and *SMARCD3* contributed most to the distinct profiles, likely affecting chromatin remodeling processes. Tumors originating from the brain or nervous tissue displayed high expression of *ACTL6B* and the cBAF-specific *DPF1* and *DPF3* contributing to a distinct cBAF profile, while tumors from kidney and thyroid had low *DPF1* and high *DPF3* expression. Other studies have demonstrated a shift in subunit paralogs from *ACTL6A* and *PHF10* to *ACTL6B*, *DPF1* and *DPF3* during the transition from neural stem cells to differentiated mature neurons [41, 42], indicating that the brain tumors retain a phenotype of differentiated cells. The brain tumors also displayed relatively high expression of *SMARCD3*. The mutually exclusive SMARCD paralogs vary between different types of cells; SMARCD3 is known to be highly expressed in muscle cells and have a specific role in the embryonic heart where it recruits SWI/SNF to heart-specific enhancers [43, 44] potentially in collaboration with DPF3 [45]. Intriguingly, a recent study identified a SMARCD3-driven neurodevelopmental epigenetic program that was hijacked in metastatic medulloblastoma, and potentially in glioblastoma [46]. Furthermore, in the same study, the transcript level of *SMARCD3* correlated with poorer prognosis in medulloblastoma. The gene expression levels of several other SWI/SNF components have prognostic values [47]. For example, an increased expression of *SMARCC2*, *SMARCD1*, *SMARCD2*, *SMARCD3*, *ACTB* and *PHF10* in renal clear cell carcinoma is associated with poor survival, while reduced expression of *SMARCA2* is associated with poor survival. In brain low grade glioma, increased expression of *ACTL6A*, *ARID1A* and *SS18*, and lower expression of *SMARCB1*, *SMARCC2*, *ACTL6B*, *DPF2* and *BCL7A* are associated with poor survival. Note that in the TCGA datasets, renal clear cell carcinoma and brain low grade glioma had the most number of genes associated with survival [48].

The distinct SWI/SNF gene expression profiles will likely result in divergent SWI/SNF functions and chromatin targeting since each subunit contribute with specific properties including chromatin- and DNA-binding regions [44]. Note that SWI/SNF components can be assembled in hundreds of theoretical combinations [49]. Although the functional consequence of distinct SWI/SNF complexes and the exact SWI/SNF-subtype compositions are complex and far from understood both in healthy and malignant tissues, diverging preferences in chromatin targeting have been reported, where cBAF complexes preferably localize to enhancers, PBAF localizes to promoters and gene bodies while GBAF localizes mostly to CTCF sites [11, 15–18]. The observed differences in SWI/SNF genes and complex subtypes may thus affect both genomic targeting, downstream transcriptional regulation and maintenance of cell identity.

In the 10 tumor types with diverging SWI/SNF expression patterns, we also observed a consistent enrichment of genes associated with PRC2, confirming an association with epigenetic regulation in these tumor types. The PRC2 complex silences genes through the H3K27me3 histone modification, and overexpression of PRC2 complexes in cancer cells can aberrantly silence tumor suppressor genes, promote cancer stem cell properties, and contribute to resistance to conventional therapies [50, 51]. Interestingly, we also found gene set enrichment for Yoshimura MAPK8 targets across the same 10 tumor types indicating that MAPK8 signaling together with SWI/SNF and PRC2 plays a pivotal role in cancer progression. MAPK8 signaling is involved in processes such as cell proliferation and survival, contributing to the aggressive nature of tumors [52].

SWI/SNF mutations are frequently observed in human cancers, many of these occur at a late state suggesting a role in tumor progression [53]. About 25% of all samples analyzed in this study had at least one mutation in a SWI/SNF gene. However, there were large variations in frequency and affected SWI/SNF components, undoubtedly connected to specific tumor types and tissue-specific functions [2, 19, 53]. SWI/SNF mutations are mostly loss-of-function mutations, i.e., the mutated SWI/SNF components are not present on protein level [54]. This results in disruption of complex integrity or paralog alterations thus affecting the chromatin affinity of the complex. The complex can also be functionally affected without impacting the structural integrity or complex composition [53, 55]. Not all types of mutations have equal consequences; while deletion, nonsense and frameshift mutations are connected to loss of function, not all missense mutations impact protein function [32]. We observed that different mutation types had distinct effects on SWI/SNF gene expression patterns which may affect transcriptional regulation differently. The fact that missense mutations commonly occur in conserved domains of SWI/SNF components, however, indicates functional consequences [19]. The key SWI/SNF genes *ARID1A* and *SMARCA4*, mutated in 8% and 4% of tumors respectively, are important for multiple biological processes. *ARID1A* acts as a tumor suppressor, playing an important role in early tumor development by dysregulation of transcription, DNA damage response and signaling pathways, such as PI3K/AKT [56]. ARID1A binds DNA via its AT-rich interacting domain and is essential for cBAF chromatin binding at enhancers and for maintaining active cell-identity enhancers [20, 44, 57]. *SMARCA4* is also a tumor suppressor gene affecting cell growth, differentiation and DNA repair processes. *SMARCA4* is commonly mutated in the conserved ATPase domain. Both truncating and missense *SMARCA4* mutations can result in loss of chromatin remodeling activity and accessibility and may hinder chromatin binding mediated by its bromodomain [58].

Notably, SWI/SNF complexes are affected in more tumors than those that contain a specific mutation in a SWI/SNF gene, as indicated by a higher frequency of SWI/SNF subunit expression loss compared to mutation frequencies in several tumor types [32]. SWI/SNF components are stabilized by complex formation. Hence, if a component that is important for the complex assembly is lost, either via mutation or suppressed expression, this may lead to disruption of complex formation and degradation of free subunits [49]. Thus, both transcriptional variations and mutations in SWI/SNF components affect SWI/SNF complex assembly and function, including genomic targeting, potentially contributing to tumor development. In our analysis, mutation in nine SWI/SNF genes showed significant association with survival in distinct tumor types. However, these survival outcomes had no correlation with the survival outcome based on SWI/SNF gene expression levels, except for *ARID1A* in brain lower grade glioma, where both low expression level and *ARID1A* mutation were associated with increased survival [47]. Furthermore, the observed increased hazard ratio for *SMARCA4* in liver hepatocellular carcinoma is in line with studies showing that certain *SMARCA4* single nucleotide polymorphisms were associated with increased risk of developing this tumor type, while loss of *SMARCA4* expression was not associated with overall survival in either the TCGA dataset or in a separate study [47, 59, 60].

Even though the frequencies of SWI/SNF mutations in different tumor types have been carefully evaluated before [2, 3, 32, 40, 53, 61], the impact on gene expression profiles were unknown. The diversity of affected SWI/SNF components and tumor types implies that there is no unique mechanism that explains tumorigenesis [53]. The general function of SWI/SNF complexes suggests that the tumor suppressive roles of SWI/SNF are likely due to aberrant transcriptional regulation but impaired DNA-damage repair also play a role [19, 20, 62]. We observed that mutations in SWI/SNF genes caused minimal effects on the expression of any SWI/SNF gene but affected downstream transcriptional profiles. In tumor types with the highest mutation frequency in SWI/SNF genes, such as colon adenocarcinoma, renal clear cell carcinoma, stomach adenocarcinoma and uterine corpus endometrial carcinoma, we observed distinct gene expression profiles in mutated samples, which were connected to epigenetic processes. For example, gene sets related to PRC2 complexes and the epigenetic mark H3K27me3 were enriched, which may be explained by the fact that dysregulated opposition of the PRC2 complex can contribute to the oncogenic function of SWI/SNF-mutated tumors [53, 63, 64]. An emerging concept is that SWI/SNF-mutant cancers have a disrupted function, especially at enhancers, as well as aberrant interactions with transcription factors, resulting in a block of development and differentiation while promoting stemness and self-renewal [20].

## Conclusions

This study underscores the major role that SWI/SNF chromatin remodeling complexes play in tumorigenesis across various tumor types. We show that certain tumor types have distinct SWI/SNF gene expression signatures and that tumors with a high frequency of SWI/SNF mutations affect downstream transcriptional profiles related to epigenetic functions. The SWI/SNF gene expression levels in tumor samples are influenced by their tissue of origin but also display extensive differences, likely due to properties acquired during tumor development. Mutations in SWI/SNF components are mostly loss-of-function, therapeutic strategies therefore rely on targeting synthetic lethalities in cancer cells, including targeting synthetic lethal relationships between SWI/SNF subunits [11, 65–67] or with PRC2 components [53, 68, 69]. Thus, certain SWI/SNF components may serve as valuable biomarkers for cancer diagnosis, prognosis, and treatment stratification. Detailed understanding of SWI/SNF gene and subtype expression opens new avenues for biomarkers and targeted therapies in cancer.

## Supplementary Information

Supplementary Table 1.

Supplementary Table 2.

Supplementary Table 3.

Supplementary Table 4.

Supplementary Table 5.

Supplementary Table 6.

Supplementary Table 7.

Supplementary Table 8.

Additional file 1.

## Data Availability

The results shown here are based upon data generated by the TCGA Research Network: https://www.cancer.gov/tcga. We utilized publicly available datasets, the batch-effect normalized mRNA expression dataset and the MC3 public version (mutation dataset), from The Cancer Genome Atlas (TCGA) Pan-Cancer (PANCAN) project, comprising 11,069 and 9,102 samples respectively, across 33 distinct tumor types. Analysis of gene expression differences in tumor and normal tissues was performed using the TNM platform.

## Abbreviations

cBAF: Canonical BAF complex
GBAF: GLTSCR1/1L BAF complex
PBAF: Polybromo BAF complex
PRC2: Polycomb repressive complex 2
SWI/SNF: SWItch/Sucrose Non-Fermentable
TCGA: The Cancer Genome Atlas
t-SNE: T-distributed stochastic neighbor embedding plot
