# Supplementary material for: Delineating SWI/SNF gene and subtype expression in cancer
Source: Epigenetics Chromatin. 2026 Aug 20;19:35. doi: 10.1186/s13072-026-00696-9 (PMC13495434; doi:10.1186/s13072-026-00696-9)
Supplement: Supplementary file 1 [file 13072_2026_696_MOESM9_ESM.pdf]

Delineating SWI/SNF gene and subtype expression in cancer

Vilma Canfjorden[1], Malin Lindén[1,2], Tobias Österlund[1,2,3], Erik Larsson[4], Anders Ståhlberg[1,2,3,5*].

[1] Sahlgrenska Center for Cancer Research, Department of Laboratory Medicine, Institute of Biomedicine, Sahlgrenska Academy, University of Gothenburg, 413 90 Gothenburg, Sweden
[2] Department of Clinical Genetics and Genomics, Sahlgrenska University Hospital, 413 45 Gothenburg, Region Västra Götaland, Sweden
[3] Wallenberg Centre for Molecular and Translational Medicine, University of Gothenburg, 413 90 Gothenburg, Sweden
[4] Department of Medical Biochemistry and Cell Biology, Institute of Biomedicine, Sahlgrenska Academy, University of Gothenburg, 413 90 Gothenburg, Sweden
[5] Science for Life Laboratory, Institute of Biomedicine, University of Gothenburg, 413 90 Gothenburg, Sweden.

Additional file 1. Supplementary information

Supplementary Figures......2

Supplementary Figure 1. Expression of SWI/SNF genes in each tumor sample......2

Supplementary Figure 2. Localization of all tumor types in the t-SNE plot......3

Supplementary Figure 3. Functional enrichment analysis for the tumor types with distinct SWI/SNF signatures......4

Supplementary Figure 4. Expression profiles of SWI/SNF genes in tumor and normal tissues in tumor types with distinct SWI/SNF profile......7

Supplementary Figure 5. Expression of selected SWI/SNF genes in tumor and normal tissues......9

Supplementary Figure 7. Types of mutations in SWI/SNF genes......12

Supplementary Figure 8. Impact of SWI/SNF mutations on the whole transcriptome......14

Supplementary Figure 9. Functional enrichment for highly mutated tumor types......15

Supplementary Figure 10. SWI/SNF mutations are associated with survival outcomes......16

Supplementary Table legends......17

Supplementary Table 1. Descriptive statistics of SWI/SNF gene expression using data from the TCGA database......17

Supplementary Table 2. Correlation of SWI/SNF genes. 17

Supplementary Table 3. Differential gene expression for the 10 tumor types with distinct SWI/SNF signature......17

Supplementary Table 4. SWI/SNF mutation information. 17

Supplementary Table 5. Categorization of mutation types in SWI/SNF genes......17

Supplementary Table 6. Gene regulation of SWI/SNF genes comparing mutated to non-mutated samples......17

Supplementary Table 7. Differential gene expression analysis comparing mutated with non-mutated samples. 17

Supplementary Table 8. Survival analysis for each SWI/SNF gene in each tumor type. 17

Supplementary Figures

Supplementary Figure 1. Expression of SWI/SNF genes in each tumor sample

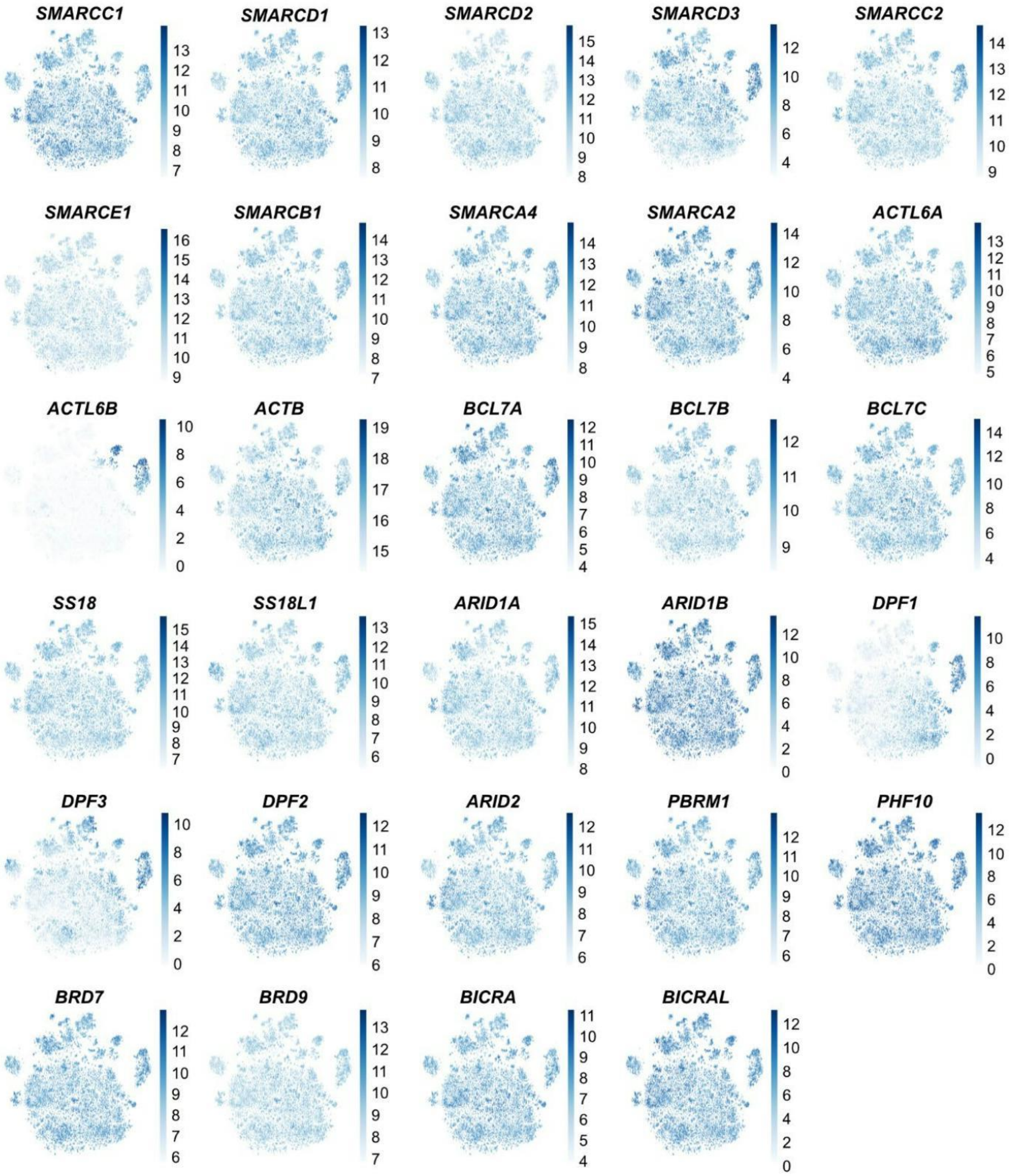

Supplementary Figure 1. Expression of SWI/SNF genes in each tumor sample. Relative gene expression of each SWI/SNF component visualized in the t-SNE plot from Figure 2A, where each tumor sample is grouped based on the expression of all 29 SWI/SNF genes. The relative expression is shown for each gene in log2-scale. Each dot is one tumor sample.

Supplementary Figure 2. Localization of all tumor types in the t-SNE plot

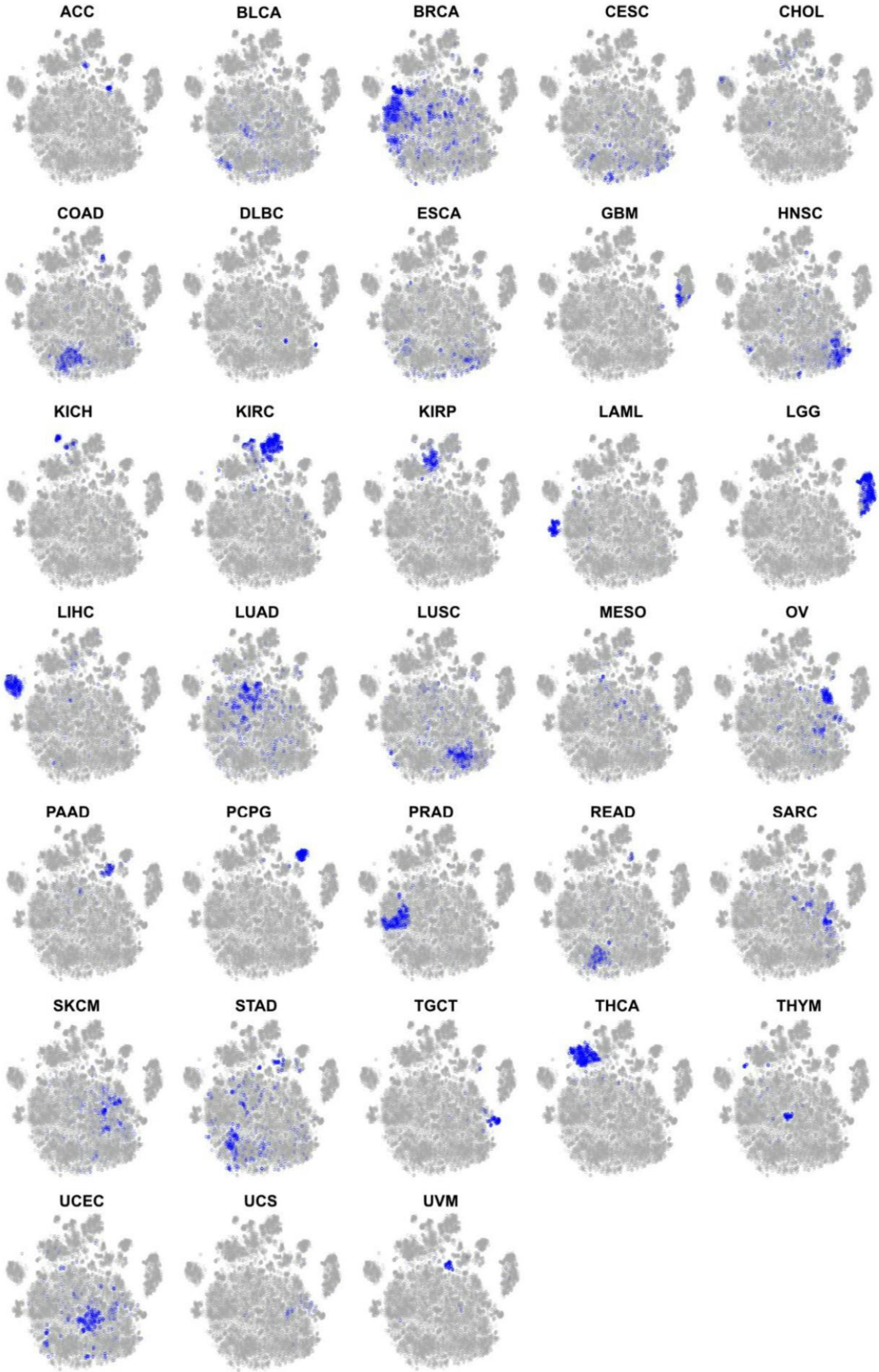

Supplementary Figure 2. Localization of all tumor types in the t-SNE plot. Visualization of the localization of each tumor type (blue) in the t-SNE plot from Figure 2A, where each tumor sample is grouped based on the expression of all 29 SWI/SNF genes. Each dot is one tumor sample.

Supplementary Figure 3. Functional enrichment analysis for the tumor types with distinct SWI/SNF signatures

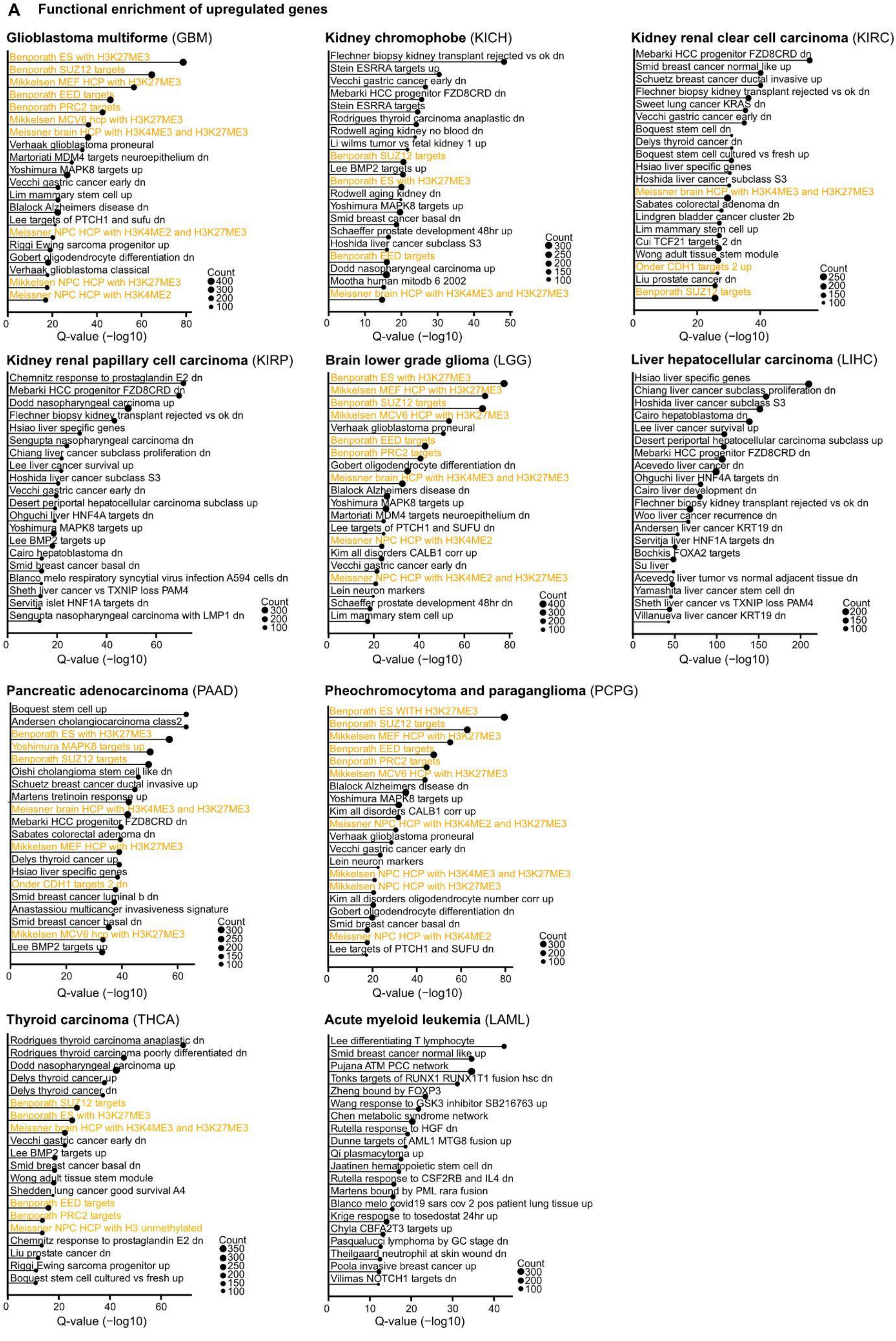

B **Functional enrichment of downregulated genes**

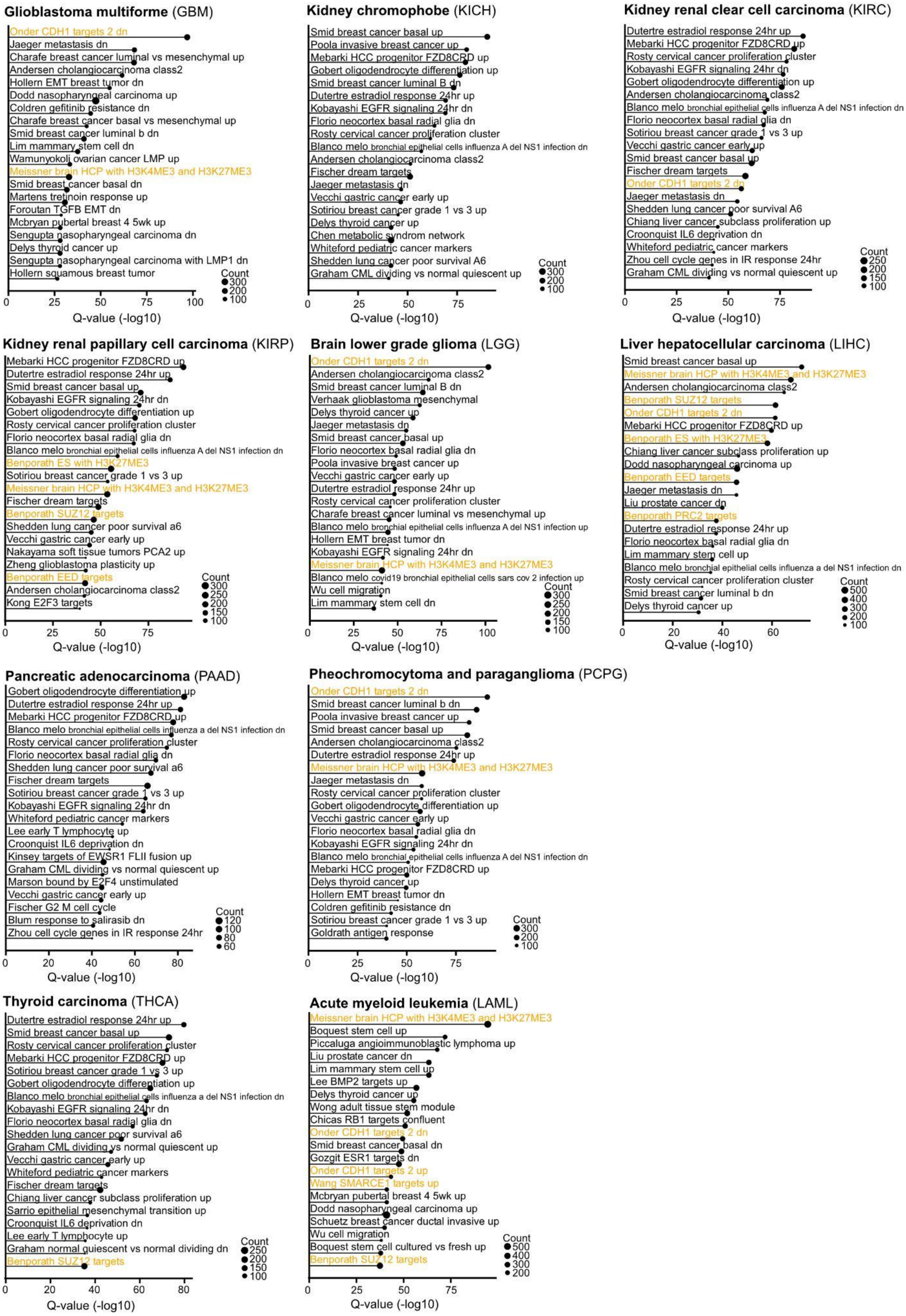

Supplementary Figure 3. Functional enrichment analysis for tumor types with distinct SWI/SNF signatures. Functional enrichment analysis using Chemical and Genetic Perturbation gene set collections for tumor entities with SWI/SNF signatures. Genes (A) upregulated or (B) downregulated in each distinct tumor type with a SWI/SNF signature compared to the remaining 23 tumor types were used, fold change cutoff > 2 and Mann-Whitney U test with Benjamini-Hochberg correction for multiple testing and adjusted $p < 0.05$. Top 20 categories are shown based on q-value. Size of dots indicates gene count. Gene sets related to epigenetic regulation are shown in orange.

Supplementary Figure 4. Expression profiles of SWI/SNF genes in tumor and normal tissues in tumor types with distinct SWI/SNF profile.

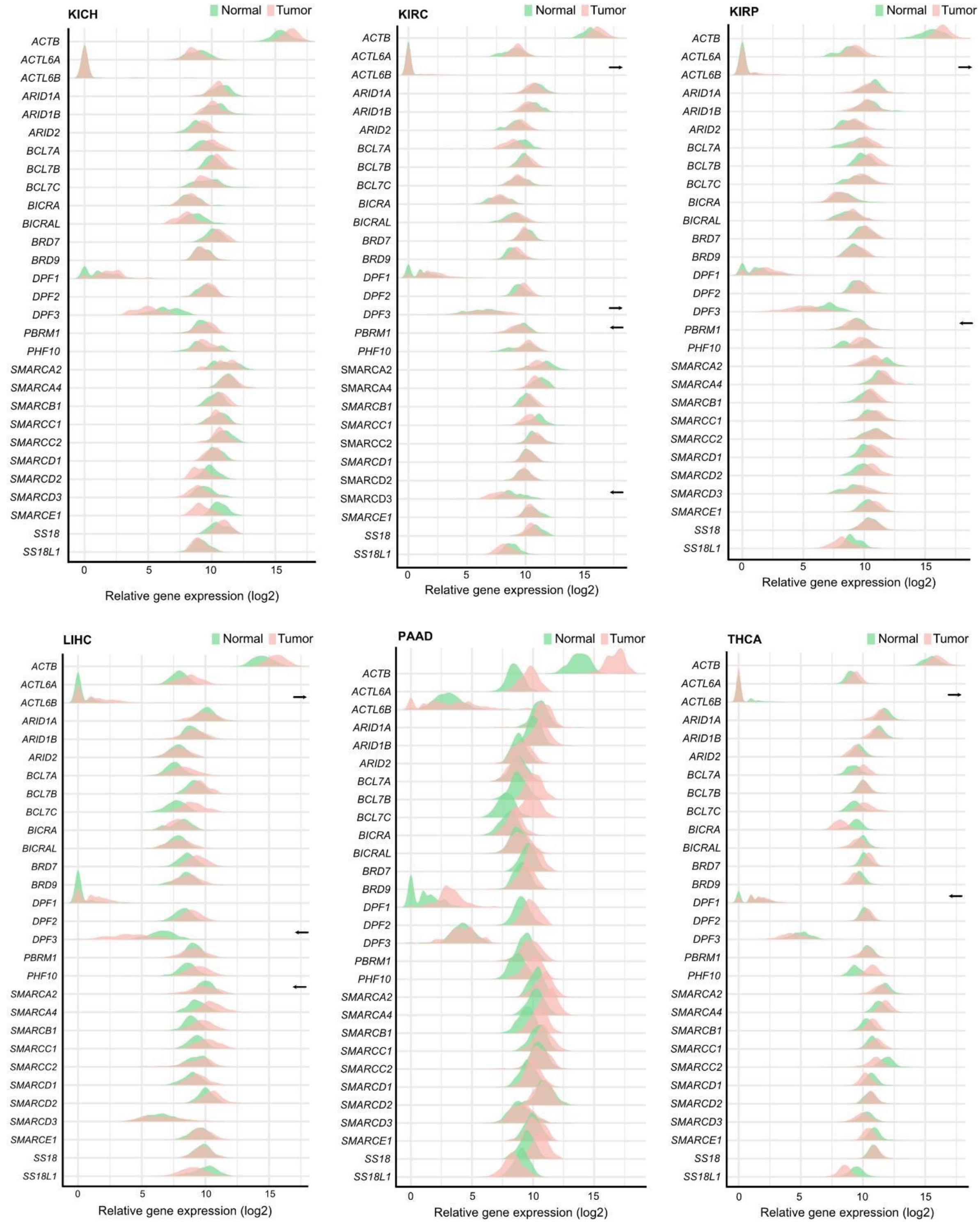

Supplementary Figure 4. Expression profiles of SWI/SNF genes in tumor and normal tissues in tumor types with distinct SWI/SNF profile. Density plots of SWI/SNF gene expression in normal and tumor tissues based on the TNMplot database. The black arrows indicate the direction of regulation of SWI/SNF subunit expression in SWI/SNF-mutated samples compared to

non-mutated samples. Only significant differences are shown based on Welch's t-test with Benjamini-Hochberg correction for multiple testing and adjusted $p < 0.05$.

Supplementary Figure 5. Expression of selected SWI/SNF genes in tumor and normal tissues.

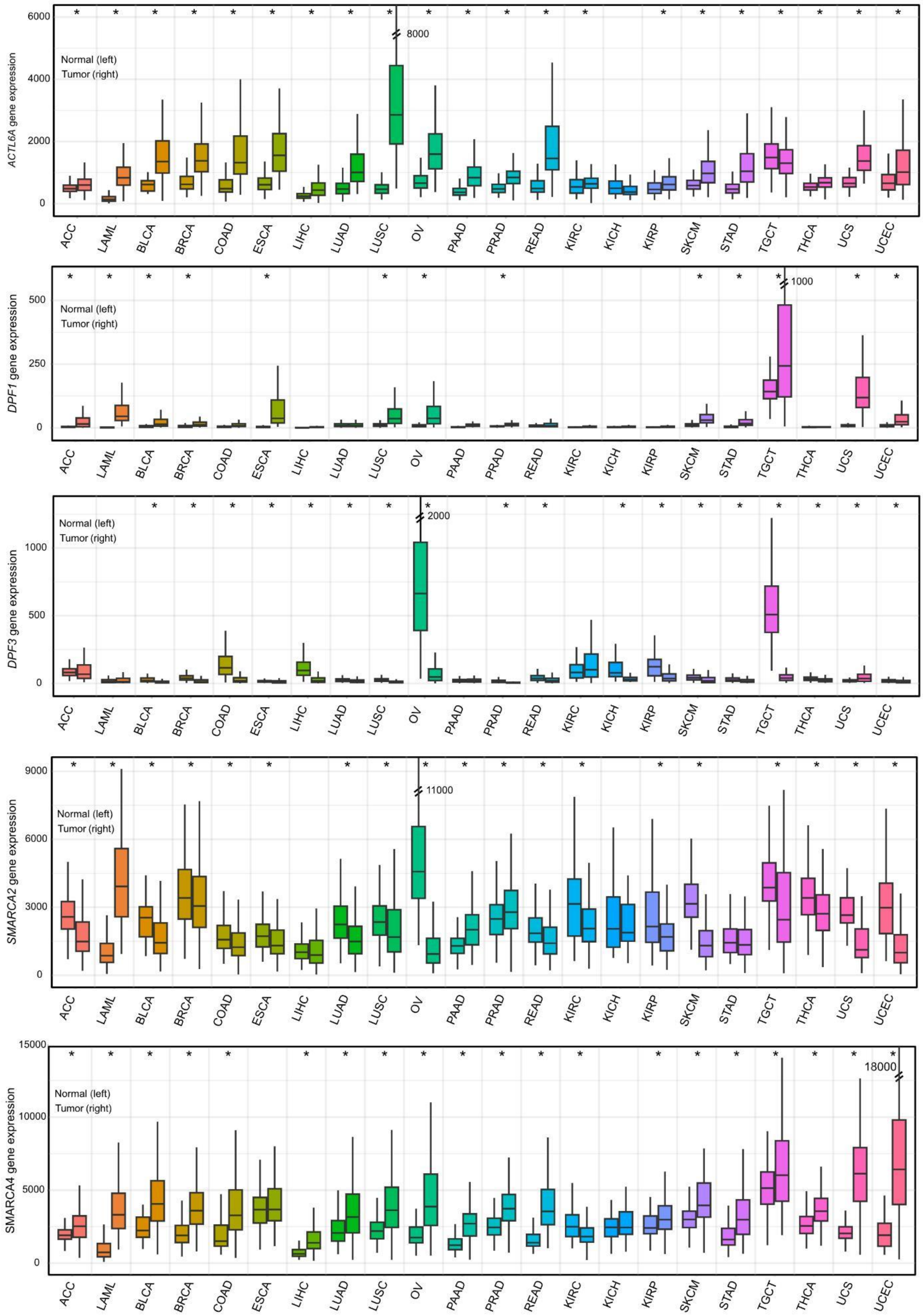

Supplementary Figure 5. Expression of selected SWI/SNF genes in tumor and normal tissues. Box plot of SWI/SNF gene expression for *ACTL6A*, *DPF1, DPF3, SMARCA2* and *SMARCA4* in normal and tumor tissues based on TNMplot database. * Significant using Mann-Whitney U test with $p < 0.05$ and a minimum expression of 10 per group. Some whiskers were cut to improve visualization, max values are indicated.

Supplementary Figure 6. SWI/SNF subtype expression

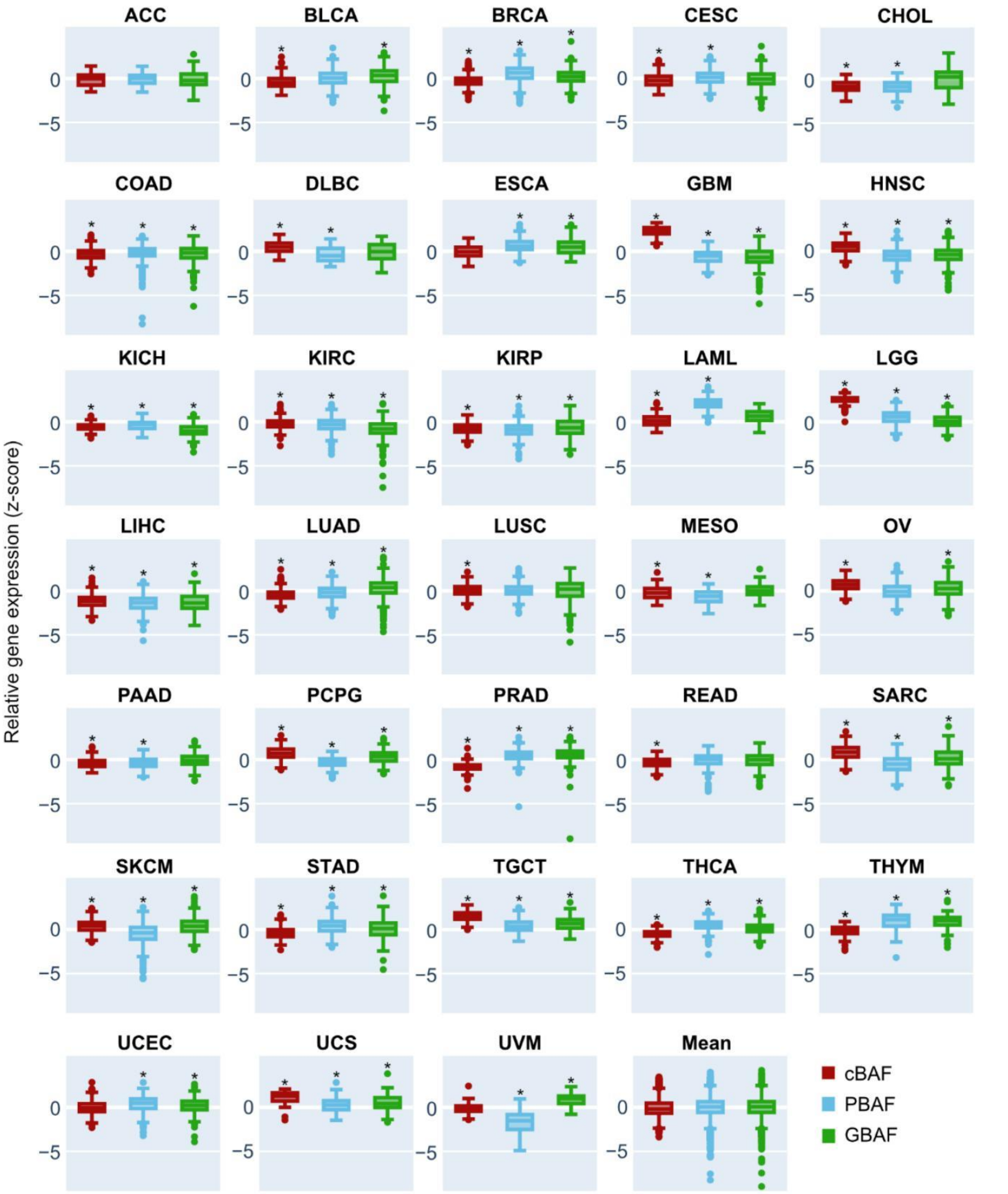

Supplementary Figure 6. SWI/SNF subtype expression. Box plot of cBAF, PBAF and GBAF expression across different tumor types. Z-scores for each tumor type are shown, where Mean represents data for all tumor types. * Significant using Student's t-test with Benjamini-Hochberg correction for multiple testing with $p < 0.05$.

Supplementary Figure 7. Types of mutations in SWI/SNF genes

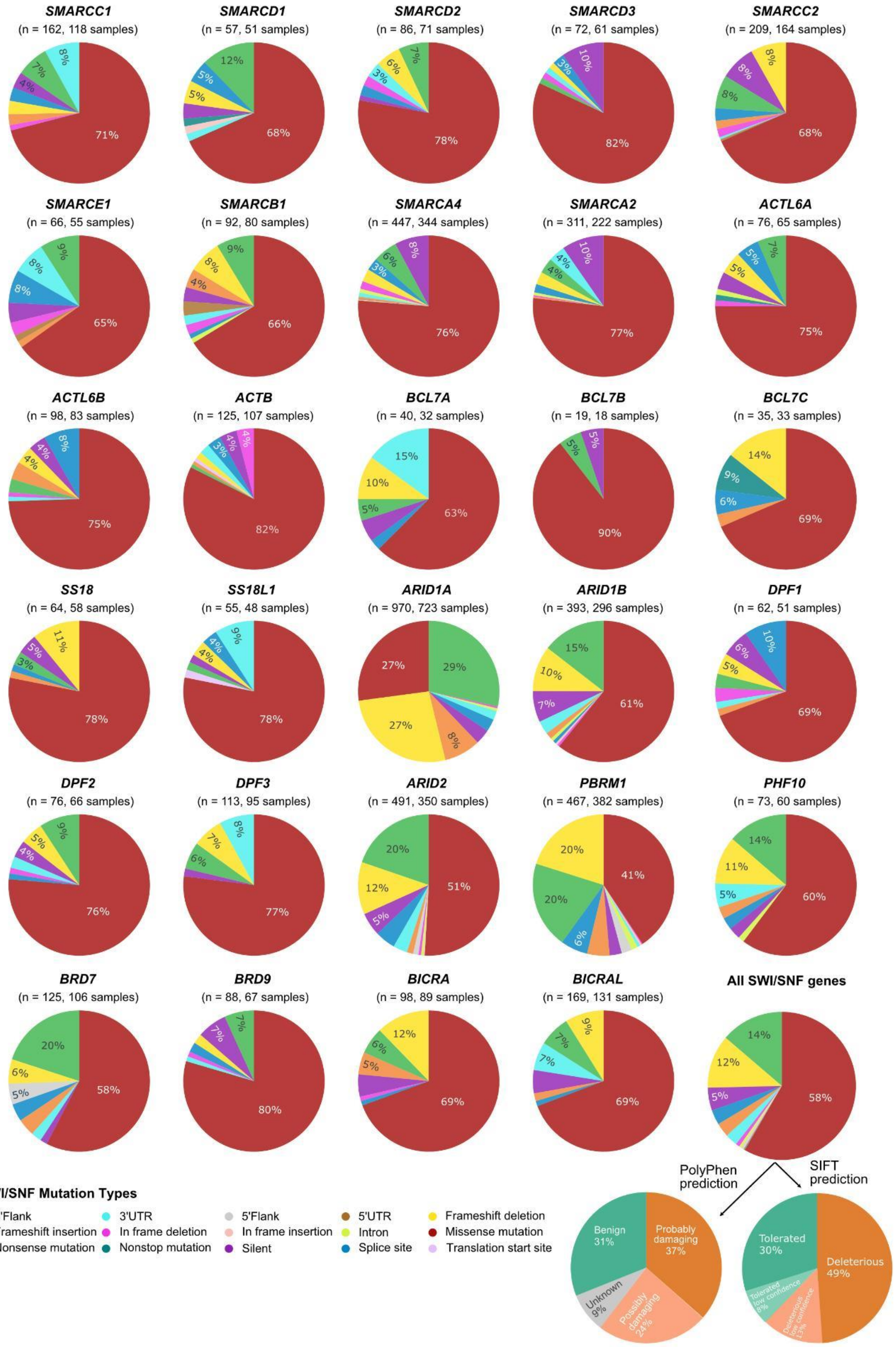

Supplementary Figure 7. Types of mutations in SWI/SNF genes. Pie charts of types of mutations for each SWI/SNF gene. The last chart shows the average mutation types for all SWI/SNF genes and indicates how many of the missense mutations are predicted to be benign, possibly damaging

or probably damaging according to PolyPhen prediction and tolerated or deleterious according to the SIFT predictor from the TCGA mutation annotation format file. Number of mutations (n) and number of samples with mutations in the specified gene are shown. Note that there can be several mutations in each gene.

Supplementary Figure 8. Impact of SWI/SNF mutations on the whole transcriptome

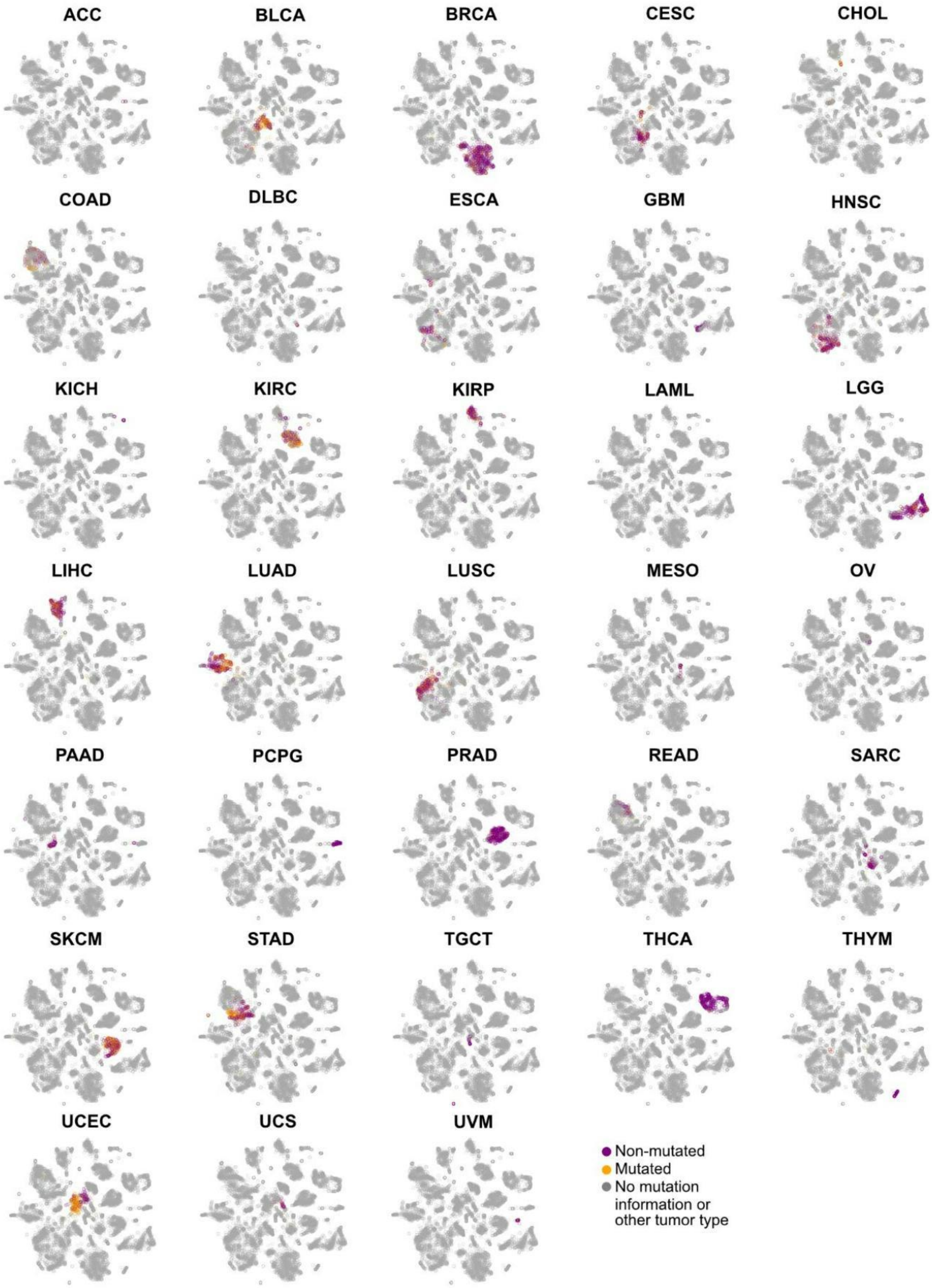

Supplementary Figure 8. Impact of SWI/SNF mutations on the whole transcriptome. t-SNE plots using the whole transcriptome with 20,533 genes and 11,069 samples. Mutated (orange) and non-mutated samples (purple) are marked for each tumor type. Grey represents either another tumor type or samples without any tumor information.

Supplementary Figure 9. Functional enrichment for highly mutated tumor types

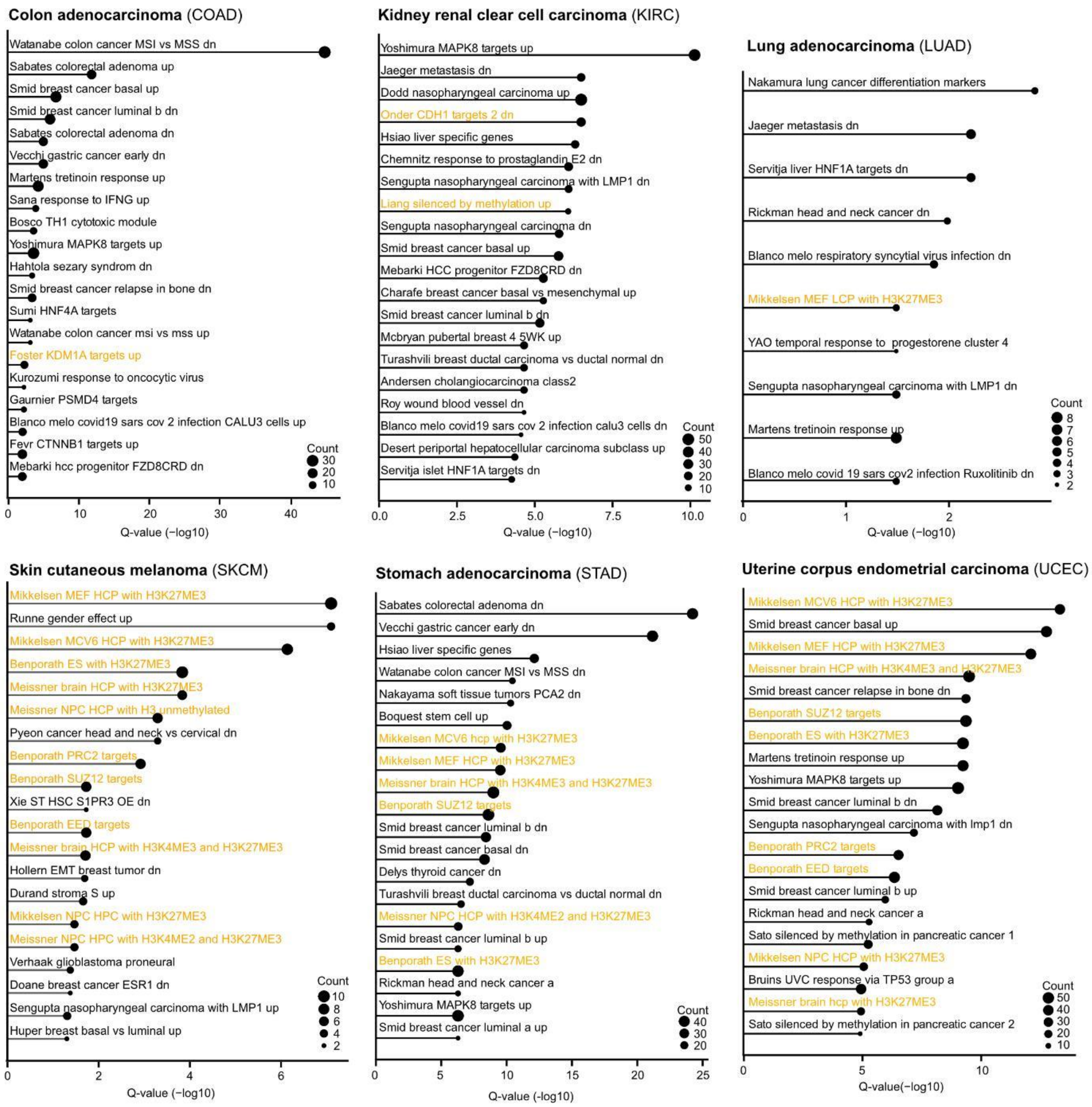

Supplementary Figure 9. Functional enrichment for highly mutated tumor types. Functional enrichment analysis using Chemical and Genetic Perturbation gene set collections for tumor entities with the highest number of regulated genes. Genes significantly regulated in mutated compared to non-mutated samples were used, based on fold change cutoff > 2 and adjusted $p < 0.05$. Top 20 significant categories are shown based on q-value. Size of dots indicates gene count. Gene sets related to epigenetic regulation are shown in orange.

Supplementary Figure 10. SWI/SNF mutations are associated with survival outcomes

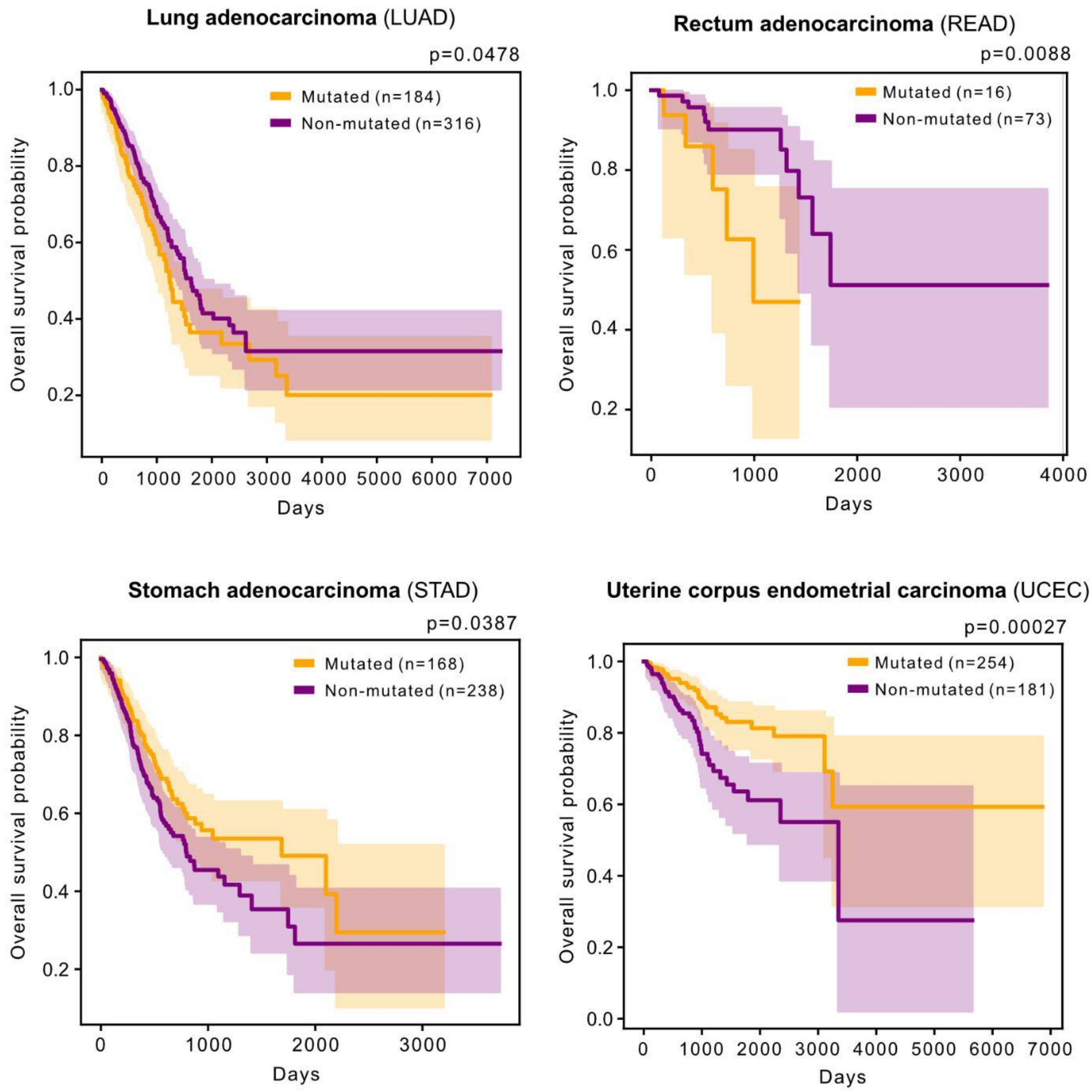

Supplementary Figure 10. SWI/SNF mutations are associated with survival outcomes. Kaplan-Meier plots visualizing overall survival of patients with tumors that have a SWI/SNF mutation compared to non-mutated. Log-rank p-values and 95% confidence interval are shown. Only tumor types with log-rank $p < 0.05$ and a minimum of 10 samples per group are shown.

Supplementary Table legends

All supplementary tables are provided as excel files (additional files 2-9). Legends are described below. (A, B etc. show different Table worksheets.)

Supplementary Table 1. Descriptive statistics of SWI/SNF gene expression using data from the TCGA database.

Supplementary Table 2. Correlation of SWI/SNF genes.
A. Spearman correlation coefficients.
B. Adjusted p-values.

Supplementary Table 3. Differential gene expression for the 10 tumor types with distinct SWI/SNF signature.

Supplementary Table 4. SWI/SNF mutation information.
A. Sample information.
B. Amount and frequency of mutated samples for each tumor type.
C. Number and frequency of samples with mutation in SWI/SNF genes.
D. Number of samples with mutation in SWI/SNF genes for each tumor type.
E. Frequency (%) of mutated SWI/SNF genes per tumor type.
F. Frequency (%) of each type of mutation in SWI/SNF genes based on classification from the TCGA mutation annotation format file.

Supplementary Table 5. Categorization of mutation types in SWI/SNF genes.

Supplementary Table 6. Gene regulation of SWI/SNF genes comparing mutated to non-mutated samples.

A. All tumor types combined.
B. For each tumor type.
C. For top 5 mutation types.

Supplementary Table 7. Differential gene expression analysis comparing mutated with non-mutated samples.

Supplementary Table 8. Survival analysis for each SWI/SNF gene in each tumor type.
